# Metal‐Responsive Up‐Regulation of Bifunctional Disulfides for Suppressing Protein Misfolding and Promoting Oxidative Folding

**DOI:** 10.1002/anie.202502187

**Published:** 2025-06-30

**Authors:** Keita Mori, Tsubura Kuramochi, Motonori Matsusaki, Yuki Hashiguchi, Masaki Okumura, Tomohide Saio, Yoshiaki Furukawa, Kenta Arai, Takahiro Muraoka

**Affiliations:** ^1^ Department of Applied Chemistry Graduate School of Engineering Tokyo University of Agriculture and Technology 2‐24‐16 Naka‐cho Koganei Tokyo 184–8588 Japan; ^2^ Department of Chemistry Massachusetts Institute of Technology 77 Massachusetts Avenue Cambridge MA 02139 USA; ^3^ Frontier Research Institute for Interdisciplinary Sciences Tohoku University 6‐3 Aramaki‐Aza‐Aoba Aoba‐ku Sendai Miyagi 980–8578 Japan; ^4^ Institute of Advanced Medical Sciences Tokushima University 3‐18‐5 Kuramoto‐cho Tokushima 770–8503 Japan; ^5^ Department of Chemistry Keio University 3‐14‐1 Hiyoshi Kohoku Yokohama Kanagawa 223–8522 Japan; ^6^ Department of Chemistry School of Science Tokai University 4‐1‐1 Kitakaname Hiratsuka Kanagawa 259–1292 Japan; ^7^ Kanagawa Institute of Industrial Science and Technology 3‐2‐1 Sakato Takatsu‐ku Kawasaki Kanagawa 213‐0012 Japan

**Keywords:** Copper ions, Disulfide bonds, Metal‐induced stress, Oxidative folding, Protein folding

## Abstract

The stress‐responsive up‐regulation process is a sophisticated biological response to maintain cellular homeostasis. In intracellular anti‐oxidant systems, the expression level of oxidoreductases is up‐regulated under oxidative stress, mitigating oxidative damage on biomolecules and enhancing protein folding capacity. Herein, inspired by the biological system, we developed a synthetic folding promotor whose reactivity is up‐regulated under stress conditions. We conjugated two metal‐binding 1,4,7,11‐tetraazacyclotetradecane (cyclam) ligands and a redox‐active disulfide to obtain cyclam‐SS, whose reactivity can be enhanced under metal‐induced stress. Metal coordination increased the redox potential of cyclam‐SS, activating it as an oxidant. While Cu^II^ ions severely hampered the oxidative folding of substrate polypeptides, cyclam‐SS exhibited bifunctional folding‐promoting properties, i) suppressing Cu^II^‐mediated misfolding and aggregation, and ii) harnessing Cu^II^ to enhance oxidative folding. Cyclam‐SS was also useful for disulfide‐bond formation to promote oxidative folding of pharmaceutical and pathological proteins, as demonstrated with proinsulin and superoxide dismutase 1 (SOD1). Furthermore, cyclam‐SS protected cultured cells from copper‐induced stress. Thus, we demonstrated the induction of the stress‐responsive up‐regulation process by a bifunctional folding promotor controlling the folding status of biologically important proteins under metal‐induced stress. The strategy of “stress‐responsive up‐regulation” could aid the development of novel synthetic materials for treating intracellular stress and related disorders.

## Graphical abstract

A stress‐responsive folding promotor was developed by conjugating metal‐binding ligands and a redox‐active disulfide. The oxidative strength of the bifunctional disulfide was enhanced by metal coordination, inhibiting protein misfolding and up‐regulating the oxidative folding capacity under metal stress. The disulfide compound also promoted oxidative folding of a pharmaceutical peptide and was used to suppress pathological protein aggregation.

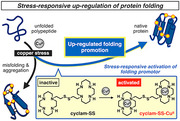

## Introduction

**Figure 1:**
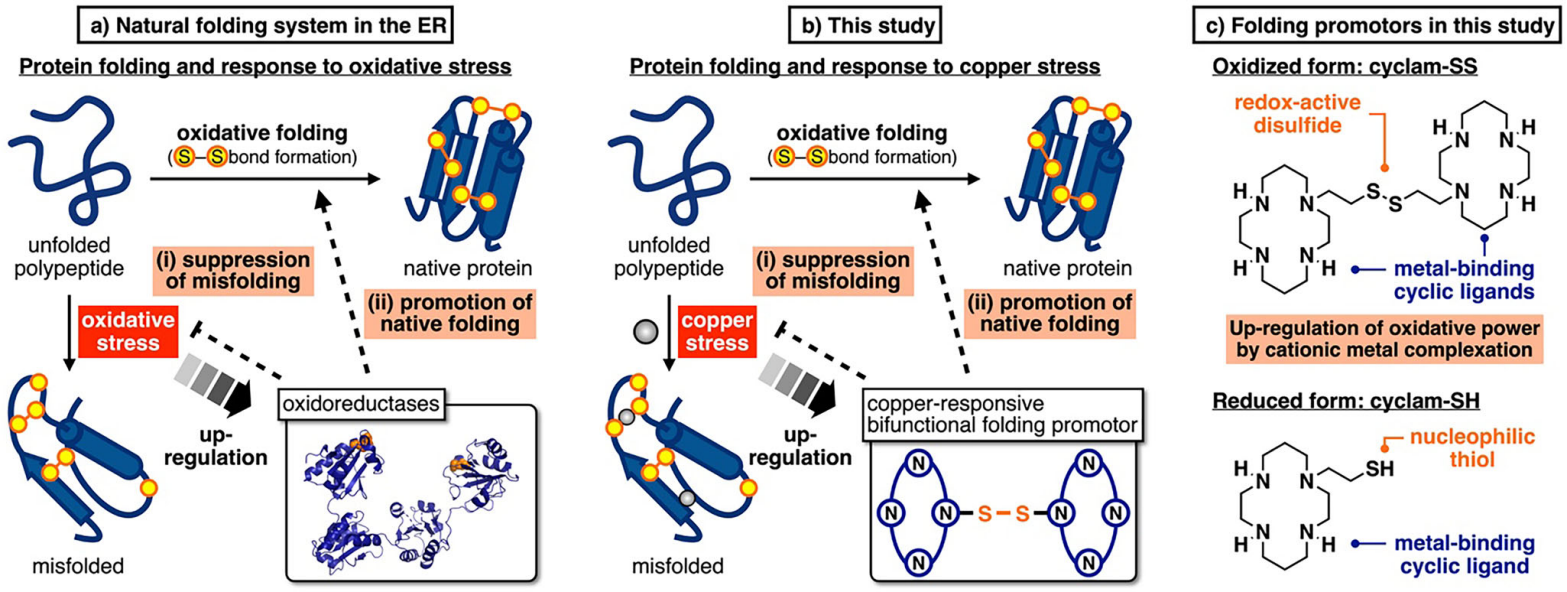
Concept behind this study. a) Natural folding systems in the endoplasmic reticulum (ER) respond to oxidative stress and promote protein folding. The expression of oxidoreductases is up‐regulated in response to oxidative stress to readjust the redox status and protein quality in the ER (PDB code: 4EL1). b) The reactivity of a copper‐responsive bifunctional folding promotor in this study is up‐regulated in response to copper stress, suppressing copper‐dependent protein misfolding and efficiently promoting native protein folding. c) Chemical structures of cyclam‐SS and cyclam‐SH, the folding promotors developed in this study.

Living cells contain sophisticated networks to deal with intracellular stress and maintain cellular homeostasis, including anti‐oxidant defense systems.^[^ ^1^, ^2^ ^]^ Typical examples are proteins containing redox‐active motifs for controlling the oxidative status of the cytosol, such as superoxide dismutase 1 (SOD1) and thioredoxin (Trx).^[^ ^3^, ^4^ ^]^ Thioredoxin‐like domains are also found in protein disulfide isomerase (PDI), a typical oxidoreductase in the endoplasmic reticulum (ER). The synthesis of disulfide‐rich secretory proteins occurs in the ER, followed by folding and transport, all of which can be adversely affected by oxidative stress due to the generation of reactive oxygen species (ROS). PDI and other proteins in this family not only aid protein folding in the ER, but also control the ER redox status. The expression levels of PDI and related proteins are regulated by inter‐organelle communication for protein quality control, called the unfolded protein response (UPR).^[^ ^5^, ^6^ ^]^ There is thus a close relationship between redox stress and protein misfolding.^[^ ^7^, ^8^ ^]^ Distortions in the ER redox balance and protein folding activate the UPR mechanism, and the expression of oxidoreductases is dynamically readjusted to i) mitigate oxidative stress to suppress protein misfolding and ii) up‐regulate the folding capacity to promote native folding (Figure 1a). These mechanisms in the ER indicate that “stress‐responsive up‐regulation” of folding capacity is key for efficiently maintaining protein quality. While many synthetic materials that mimic natural protein functions have been reported,^[^ ^9^, ^10^ ^]^ it has remained unexplored to develop enzyme‐mimetic molecules with stress‐responsive up‐regulation properties. In this study, we focused on intracellular metal‐induced stress and developed a bifunctional compound which up‐regulates its own folding capacity in response to stress‐causing metal ions, in order to i) suppress metal‐dependent protein misfolding and aggregation, and ii) promote native folding (Figure 1b).

Metal ions are indispensable in many biological events,^[^ ^11^, ^12^ ^]^ such as the metal‐dependent stabilization of biomolecular assemblies^[^ ^13^, ^14^ ^]^ and metal‐mediated signal transduction.^[^ ^15^, ^16^ ^]^ In particular, transition metal ions often bind to proteins to stabilize them and allow catalytic activity.^[^ ^17^ ^]^ However, even biologically essential metal ions become cytotoxic if their homeostasis is imbalanced, causing cellular stress and severe diseases.^[^ ^18^, ^19^, ^20^ ^]^ Copper ions, while playing critical roles due to their binding features and redox activity,^[^ ^21^, ^22^, ^23^ ^]^ can be toxic at elevated levels by non‐specifically associating with proteins to induce misfolding and aggregation, and by triggering redox reactions to cause oxidative damage of proteins and other biomolecules.^[^ ^24^, ^25^, ^26^, ^27^, ^28^ ^]^ Aggregation of several pathological proteins is reportedly enhanced in the presence of copper ions, suggesting a relationship between “copper stress” and neurodegenerative disorders.^[^ ^29^, ^30^, ^31^ ^]^ Metal‐related diseases are treated using metal chelators to scavenge and remove target metal ions.^[^ ^32^, ^33^, ^34^ ^]^ However, it is also important to understand how to circumvent metal‐induced promiscuous oxidation and pathological misfolding of proteins. A methodology to promote native protein folding and to maintain protein quality under metal‐induced stress is thus required.

Proteins containing cysteine residues, many of which are bridged through specific pairs of disulfide (S─S) bonds during the folding process, are severely affected by copper stress due to the metal‐binding and redox‐active features of the cysteines. The folding reaction accompanied by S─S bond formation is intrinsically oxidative process, namely oxidative protein folding.^[^ ^35^, ^36^, ^37^, ^38^ ^]^ Many secretary proteins, membrane proteins, and antibodies contain S─S bonds, leading to studies on the artificial promotion of oxidative protein folding, with the intent of enhancing the production of functional proteins and inhibiting pathological misfolding. PDI contains a CGHC motif as a redox‐active center that can form intramolecular S─S bonds for efficient oxidation reactions.^[^ ^39^, ^40^ ^]^ The thiol groups in the reduced form of PDI are highly nucleophilic due to the imidazolyl group on the neighboring histidine, promoting isomerization of the S─S bonds to assist the folding process. The chemical properties of PDI have inspired the design of various thiol and disulfide compounds as synthetic folding promotors, including their diselenide derivatives.^[^ ^41^, ^42^, ^43^, ^44^ ^]^ As previously demonstrated, the molecular moieties adjacent to the redox‐active centers affect the oxidative folding capacity and functionality of the compound.^[^ ^45^, ^46^, ^47^, ^48^ ^]^ We recently revealed that the introduction of cationic moieties enhances the oxidative power of disulfide compounds,^[^ ^49^ ^]^ inspiring us to investigate cationic metal complexation to trigger and promote oxidative protein folding. We anticipated that ligand‐modified disulfide compounds can bind stress‐causing metal cations and enhance their own reactivity due to the increased positive charge. Herein, we conjugated a metal‐binding cyclic ligand 1,4,7,11‐tetraazacyclotetradecane (cyclam) and a redox‐active disulfide group to synthesize cyclam‐SS as a folding‐promoting compound whose oxidative strength and folding capacity can be enhanced under copper stress for up‐regulating responses (Figure 1b,c). A reduced analog, cyclam‐SH, was designed as another folding promotor that is nucleophilic to promote shuffling of the S─S bonds during oxidative folding.

## Results and Discussion

### Synthesis of Cyclam‐SS

The cyclam‐tethered disulfide cyclam‐SS and its reduced analog cyclam‐SH (Figure 1c) were synthesized from commercially available 2‐hydroxyethyldisulfide (**1**) and cyclam (**2**). The two hydroxy groups of **1** and the three amino groups of **2** were protected with Tosyl and Boc moieties, respectively, to obtain compounds **3** and **4**, according to previous reports.^[^ ^50^, ^51^ ^]^ The conjugation of **3** and **4** in the presence of potassium carbonate gave disulfide‐bridged cyclam with Boc‐protecting groups (**5**). A deprotection reaction under acidic conditions yielded the target compound cyclam‐SS (**6**). Cyclam‐SH (**7**) was synthesized by cleaving the S─S bond of cyclam‐SS with dithiothreitol (DTT). Synthetic procedures and characterization results for the compounds are shown in Supporting Information.

### Cu^II^‐Dependent Changes in the Oxidative Power of Cyclam‐SS

We designed cyclam‐SS as a synthetic folding promotor whose ability to promote oxidative folding is enhanced under copper stress. We first examined the complexation of cyclam‐SS with copper ions. Cyclam ligands form stable complexes with a broad range of transition metal ions and especially show remarkable affinity toward Cu^II^ ions (log *K* = 26.5)^[^ ^52^ ^]^ because the cyclam structure supports the tetracoordinated square‐planar geometry typical of Cu^II^ complexes. We analyzed the changes in UV absorbance of cyclam‐SS upon the addition of Cu^II^ ions to evaluate its Cu^II^ complexation. Essentially no UV absorption was observed in the absence of Cu^II^ ions, whereas the addition of Cu^II^ ions significantly enhanced the absorption at 274 nm due to ligand‐to‐metal charge transfer (LMCT), as reported in previous studies of cyclam–Cu^II^ complexes,^[^ ^53^ ^]^ together with minor absorption around 600 nm derived from the d–d transition of Cu^II^ (Figure S1a). This absorbance change was similar to that of cyclam alone and the mixture of cyclam and 2‐hydroxyethyldisulfide (Figure S1b). This result indicated that the covalent linkage of cyclam‐SS between cyclam and the disulfide linker does not critically diminish its Cu^II^ affinity, suggesting its Cu^II^‐binding behaviors similar to cyclam under the solution condition tested in this study. The titration of Cu^II^ ions into a solution of cyclam‐SS increased the absorbance at 274 nm almost linearly in the range of [Cu^II^]/[cyclam‐SS] = 0 to 1.0. The trend changed when more Cu^II^ ions were added (Figure S1c), suggesting that the first Cu^II^ ion was quantitatively incorporated and the second Cu^II^ binds with lower affinity to cyclam‐SS, possibly because the binding of two Cu^II^ ions causes severe electrostatic repulsion between the two Cu^II^ centers. We thus used 1 equiv. of Cu^II^ ions to evaluate the reactivity of cyclam‐SS and to operate the protein folding reactions with cyclam‐SS.

We conducted redox potential measurements to study the effect of Cu^II^ ions on the oxidative power of cyclam‐SS (Table 1). Oxidative protein folding is driven by the formation of S─S bonds, and thus the oxidative strength of a disulfide compound greatly impacts its utility as a folding promotor. The redox potential *E*°′ indicates the oxidative strength of a compound; the higher the value of *E*°′, the better the oxidant. The *E*°′ value of cyclam‐SS was −231 ± 0.8 mV, which is higher than that of glutathione (GSSG, *E*°′ = −256 mV),^[^ ^43^ ^]^ an intracellular redox‐active compound. This remarkable oxidative power of cyclam‐SS is likely due to the inherent cationic charges on the cyclam ligands at neutral pH, given the reported p*K*_a_ values of cyclam (p*K*_a1_ = 11.5, p*K*_a2_ = 10.2, p*K*_a3_ = 1.6, p*K*_a4_ = 0.9)^[^ ^54^ ^]^ and tetra‐*N*‐methylated cyclam (p*K*_a1_ = 9.3, p*K*_a2_ = 9.0, p*K*_a3_ = 2.6, p*K*_a4_ = 2.2).^[^ ^55^ ^]^ Electrostatic repulsion between the positive charges can destabilize the disulfide and promote its conversion to the thiol form, improving its performance as an oxidant. The redox potential of 2‐hydroxyethyldisulfide as a control was reported to be −252 mV,^[^ ^56^ ^]^ and it was not significantly affected by the addition of cyclam (−250 ± 8.8 mV, Table S1). This result indicated the importance of the covalent linkage between the cyclam rings and the disulfide linker, validating the molecular design in this study. The presence of 1 equiv. of Cu^II^ ions further enhanced the redox potential of cyclam‐SS (*E*°′ = −214 ± 6.4 mV). This Cu^II^‐dependent increase in *E*°′ can be due to the additional positive charges on cyclam‐SS provided by Cu^II^ complexation. The addition of Ni^II^ ions enhanced the oxidative strength of cyclam‐SS (*E*°′ = −211 ± 2.9 mV) similar to that of Cu^II^ ions whereas Zn^II^ ions provided no significant effect (*E*°′ = −230 ± 3.1 mV). These results reflect the affinity of the metal ion toward the cyclam ligand (log *K*_1_ values of cyclam–metal complexes; Cu^II^: 26.5, Ni^II^: 22.2, Zn^II^: 15.5),^[^ ^52^ ^]^ indicating that stable metal complexation is important for a metal‐dependent increase in the redox potential. Considering the reported binding constant of tetra‐*N*‐methylated cyclam with Cu^II^ ions (log *K*_1_ = 18.3),^[^ ^55^ ^]^ it can be speculated that cyclam‐SS, which has *N*‐alkyl modification, binds to Cu^II^ strongly enough to show quantitative complexation at micromolar concentrations. We thus confirmed that the oxidative ability of cyclam‐SS is strengthened by complexation with Cu^II^ ions.

**Table 1:** Redox potential values of cyclam‐SS and related compounds.

Compound	Redox potential (*E*°′) / mV^a)^
cyclam‐SS	−231 ± 0.8
cyclam‐SS–Cu^II^	−214 ± 6.4
cyclam‐SS–Ni^II^	−211 ± 2.9
cyclam‐SS–Zn^II^	−230 ± 3.1
glutathione (GSSG)	−256 ± 5^b)^

^a)^Error values indicate the means ± SEM of three independent experiments.

^b)^Ref. 43.

We also studied the chemical properties of the reduced analog cyclam‐SH. Nucleophilicity is an important parameter when discussing the activity of a compound for promoting disulfide‐shuffling reactions. Here we evaluated the nucleophilicity by measuring the p*K*_a_ value of cyclam‐SH based on the pH‐dependent deprotonation of the thiol group and the resultant absorbance change at 240 nm. The p*K*_a_ value of the thiol group on cyclam‐SH was 8.48 ± 0.05 (Figure S2), which is lower than that of glutathione (GSH, p*K*_a_ = 9.15 ± 0.04),^[^ ^57^ ^]^ highlighting the superior reactivity of cyclam‐SH as a nucleophile. We then examined the effects of Cu^II^ ions on the nucleophilicity of cyclam‐SH, but measuring the p*K*_a_ was difficult in the presence of Cu^II^ ions because the absorption of the cyclam–Cu^II^ complex overlapped that of thiolate anions, obscuring changes in the absorbance. Therefore, the nucleophilicity of cyclam‐SH was studied based on a disulfide‐shuffling reaction with 5,5′‐dithiobis‐(2‐nitrobenzoic acid) (DTNB). Thiol compounds can attack the S─S bond of DTNB to yield the SH form of DTNB, which can be detected by absorbance at 412 nm. As shown in Figure S3, cyclam‐SH reacted with DTNB more rapidly than did GSH, consistent with the p*K*_a_ values discussed above. However, in the presence of Cu^II^ ions, the absorbance at 412 nm recovered only marginally, indicating that the disulfide‐shuffling reaction was severely hampered by Cu^II^ ions, perhaps because the thiolate group on cyclam‐SH coordinates to a Cu^II^ ion and is inactivated as a nucleophile. These results revealed that Cu^II^ ions can especially enhance the oxidative strength of cyclam‐SS.

### Oxidative Folding of Bovine Pancreatic Trypsin Inhibitor by Cyclam‐SS

**Figure 2:**
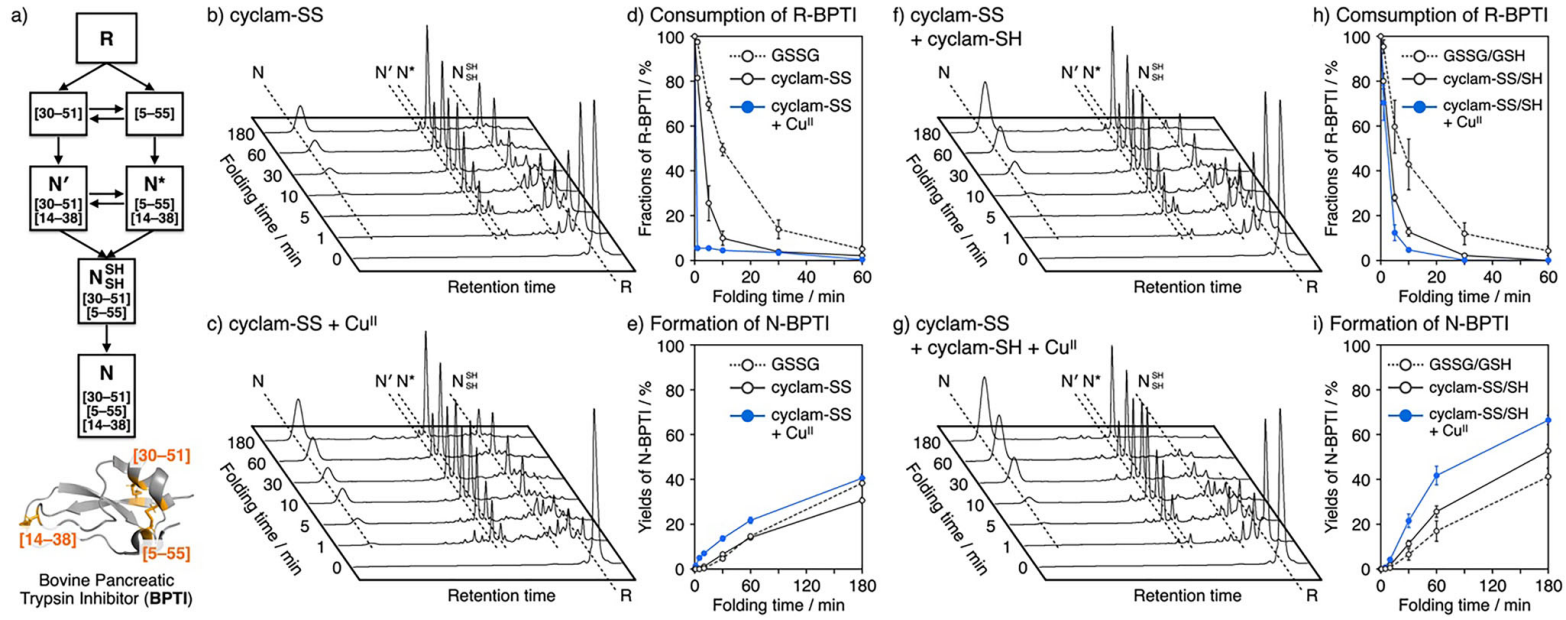
Oxidative folding of BPTI driven by cyclam‐SS. a) Folding pathway of BPTI. b), c) Time‐course reverse‐phase HPLC (RP‐HPLC) analysis of the oxidative folding of BPTI by cyclam‐SS b) in the absence and c) in the presence of Cu^II^ ions. d), e) Time‐course plots of the fractions of d) reduced BPTI (R‐BPTI) and e) native BPTI (N‐BPTI) during BPTI folding by cyclam‐SS. [BPTI] = 30 µM, [SS compounds] = 90 µM, [CuCl_2_] = 0 or 90 µM. [urea] = 300 mM, 50 mM Tris–HCl (pH 7.5), 300 mM NaCl, 30 °C. f), g) Time‐course RP‐HPLC analysis of the oxidative folding of BPTI by cyclam‐SS and cyclam‐SH f) in the absence and g) in the presence of Cu^II^ ions. h), i) Time‐course plots of the fractions of h) R‐BPTI and i) N‐BPTI during BPTI folding by cyclam‐SS and cyclam‐SH. [BPTI] = 30 µM, [SS compounds] = 90 µM, [SH compounds] = 900 µM, [CuCl_2_] = 0, or 180 µM. [urea] = 300 mM, 50 mM Tris–HCl (pH 7.5), 300 mM NaCl, 30 °C. The eluent buffers for the HPLC analyses: water (containing 0.05% TFA) and CH_3_CN (containing 0.05% TFA) with a linear gradient; flow rate: 1.0 mL min^−1^; detection wavelength: 229 nm; column temperature: 50 °C. Error bars indicate the means ± SEM of three independent experiments.

Encouraged by the remarkable redox activity and Cu^II^‐responsiveness of cyclam‐SS, we examined oxidative protein folding driven by cyclam‐SS using bovine pancreatic trypsin inhibitor (BPTI) as a model substrate. Native BPTI contains three S─S bonds, between C5–C55, C14–38, and C30–51. The folding of BPTI from the reduced form (R‐BPTI) to the native form (N‐BPTI) involves three quasi‐native intermediates, N′, N*, and $N_{\mathrm{SH}}^{\mathrm{SH}}$,^[^ ^58^ ^]^ (Figure 2a) and can be monitored by reverse‐phase high performance liquid chromatography (RP‐HPLC). R‐BPTI (30 µM) was treated with 3 equiv. of cyclam‐SS (90 µM), which is quantitative to the three S─S bonds in N‐BPTI, to promote oxidative folding. HPLC analysis revealed that essentially no R‐BPTI remained after 10 min and that N‐BPTI was gradually formed along with other intermediates, providing a 31% yield of N‐BPTI after 3 h of incubation (Figure 2b). Cyclam‐SS promoted the BPTI folding better than the mixture of cyclam and 2‐hydroxyethyldisulfide (Table S2), confirming the importance of the covalent linkage between cyclam and the disulfide linker as suggested by the comparison of the redox potential in Table S1. The presence of Cu^II^ ions (90 µM) in addition to cyclam‐SS accelerated BPTI folding compared to in the absence of Cu^II^, providing a yield of 41% N‐BPTI after 3 h (Figure 2c). Time‐course changes of the R‐BPTI and N‐BPTI fractions are shown in Figure 2d,e. Cyclam‐SS consumed R‐BPTI faster than did GSSG, indicating that cyclam‐SS accelerated the oxidation reaction, consistent with its redox potential (Table 1). The rate of R‐BPTI disappearance (*k*_red_) in the BPTI folding by cyclam‐SS and Cu^II^ (*k*_red_ = 2.92 min^−1^) was approximately 11‐fold higher than the Cu^II^‐free conditions (0.263 min^−1^) and showed 42‐fold enhancement compared with the reaction using GSSG instead (0.0699 min^−1^). This indicates the significant acceleration of the S─S introduction process (Figure S4a). The rate of N‐BPTI formation (*k*_nat_) was also enhanced by mixing cyclam‐SS and Cu^II^ compared with other conditions (Figure S4c). It was thus indicated that the complexation of cyclam‐SS and Cu^II^ ions promotes S─S bond formation, likely due to the improved oxidation ability of cyclam‐SS following Cu^II^ complexation, as demonstrated by the data shown in Table 1.

With a view to further accelerating the folding process, we performed BPTI folding experiments in the presence of the folding promoters cyclam‐SS and its reduced analog, cyclam‐SH (Figure 1c). Thiol compounds can promote the shuffling of S─S bonds by nucleophilic attack, and so the addition of cyclam‐SH might enhance interconversion between the folding intermediates, providing a higher N‐BPTI yield. Titration of cyclam‐SH into a solution of R‐BPTI and cyclam‐SS improved the yield of N‐BPTI (Figure S5). The increase in the efficiency of the N‐BPTI formation was converged at 900 µM cyclam‐SH in the series of the concentrations tested, resulting in 53% yield after 3 h (Figure 2f). We then examined BPTI folding in the presence of Cu^II^ ions. When the Cu^II^ concentration was gradually increased, the yield of N‐BPTI was enhanced in a Cu^II^‐dependent manner and was essentially maximum at 180 µM Cu^II^ (Figure S6). The addition of 180 µM Cu^II^ to cyclam‐SS and cyclam‐SH provided N‐BPTI in 66% yield after 3 h of incubation (Figure 2g). Time‐course plots and the rate analysis of the R‐BPTI fractions showed that the oxidation reactions were accelerated in the presence of cyclam‐SS and cyclam‐SH compared to a mixture of oxidized and reduced glutathione (GSSG and GSH, respectively), and were further promoted by the presence of Cu^II^ ions (Figures 2h and S4b). N‐BPTI yields were increased by replacing glutathione with cyclam‐SS and cyclam‐SH, and further improved in the presence of Cu^II^ ions due to the Cu^II^‐responsive activation of cyclam‐SS (Figures 2i and S4d). We therefore demonstrated that cyclam‐SS is a folding promotor whose activity is up‐regulated by the presence of copper ions, accelerating oxidative protein folding.

### Investigation of the Interaction Between Cyclam‐SS and BPTI by NMR Measurements

**Figure 3:**
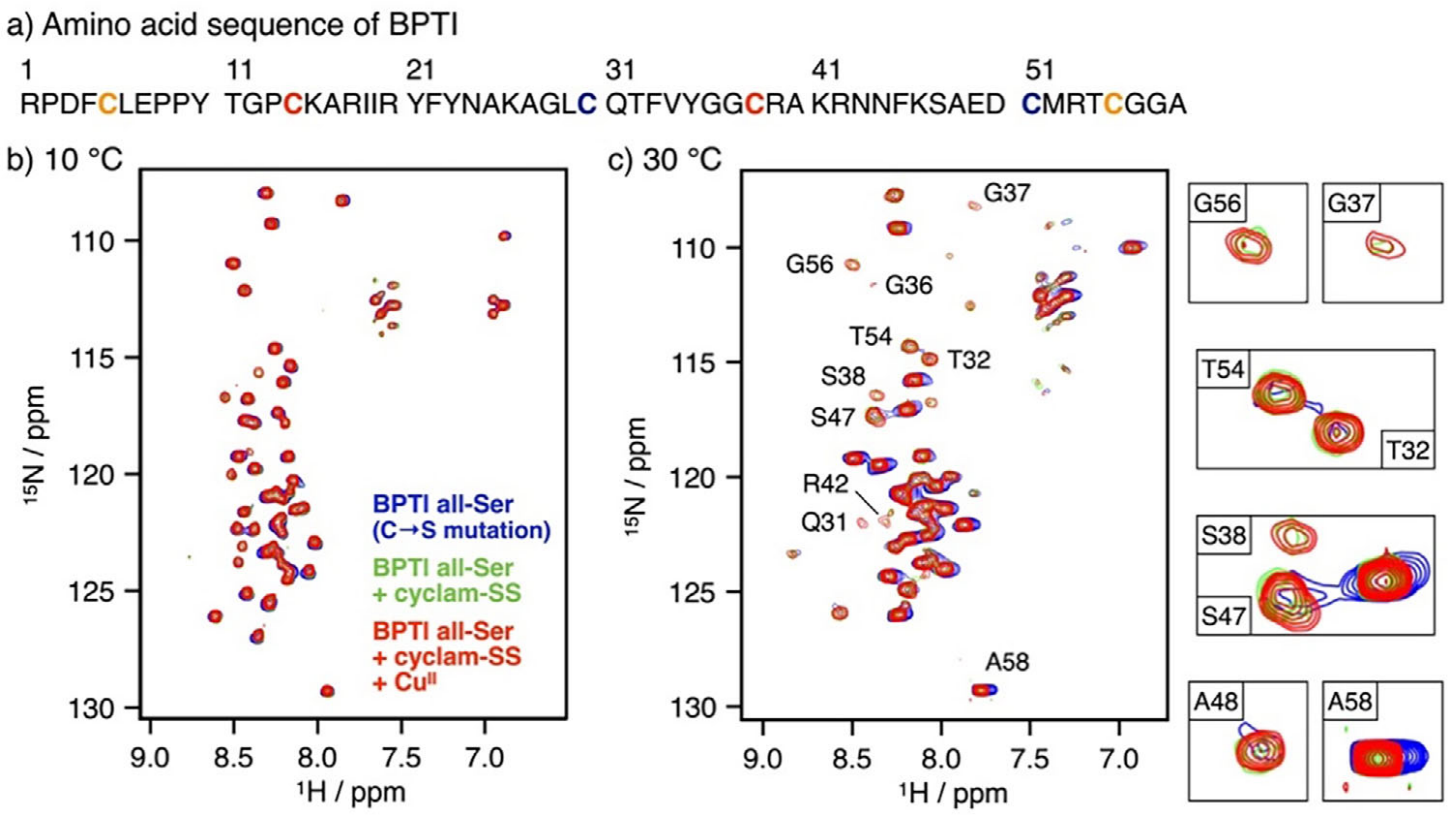
^1^H–^15^N 2D NMR analysis of ^15^N‐labeled denatured BPTI (BPTI All‐Ser). a) Amino acid sequence of BPTI. For the NMR analysis, all the cysteine residues were mutated to serine. b), c) ^1^H–^15^N correlation SOFAST‐HMQC spectra of ^15^N BPTI All‐Ser in the absence and in the presence of cyclam‐SS. Measurements were conducted at b) 10 °C and c) 30 °C. Magnified views of several signals with characteristic differences at 30 °C are shown. [^15^N BPTI All‐Ser] = 100 µM, [cyclam‐SS] = 0 or 1 mM, [CuCl_2_] = 0 or 1 mM, 50 mM HEPES (pH 7.0), 10 v/v% D_2_O, 500 MHz.

The promotion of BPTI folding by cyclam‐SS discussed above encouraged us to further study the interaction between cyclam‐SS and BPTI to clarify the molecular mechanisms underlying folding promotion. Considering that cyclam‐SS promoted the rapid introduction of S─S bonds into R‐BPTI, we focused on the molecular interactions at the early stage of the folding reaction using NMR analyses of reduced and denatured BPTI in a completely random coil structure (Figure 3). We prepared ^15^N‐labeled BPTI in which all the amide nitrogen atoms are labeled with ^15^N, so that signals from different amino acid residues could be distinguished by ^1^H─^15^N 2D NMR measurements for residue‐specific assessment of intermolecular interactions.^[^ ^59^, ^60^ ^]^ In addition, all the cysteine residues were mutated to serine to provide denatured BPTI (BPTI all‐Ser), which is free from S─S bond formation (Figure 3a). ^1^H─^15^N SOFAST‐HMQC NMR spectra of ^15^N‐labeled BPTI all‐Ser showed no significant signal changes at 10 °C even after the addition of cyclam‐SS or cyclam‐SS–Cu^II^ (Figure 3b), whereas after increasing the measurement temperature to 30 °C, the NMR spectra changed significantly. In the absence of cyclam‐SS, several signals broadened at 30 °C, indicating the promotion of intermolecular aggregation of ^15^N‐labeled BPTI all‐Ser at this higher temperature (Figure 3c). In contrast, the addition of cyclam‐SS inhibited the signal broadening and kept those signals sharp at 30 °C. These results suggested that cyclam‐SS weakly interacts with BPTI to suppress its intermolecular aggregation. Because GSSG showed only minor effects compared with cyclam‐SS at 30 °C (Figure S7), the cyclam ligand apparently plays a key role in the interaction with BPTI. Further increasing the concentration of cyclam‐SS resulted in the perturbation of several signals at 10 °C (Figure S8), showing the interaction of cyclam‐SS with specific amino acid residues in BPTI all‐Ser. Cyclam‐SS thus can promote the native folding of BPTI by suppressing intermolecular aggregation to enhance intramolecular noncovalent interaction and S─S bond formation. Furthermore, the effect of cyclam‐SS was unchanged even in the presence of Cu^II^ ions, indicating that Cu^II^ binding strengthened the oxidative ability of cyclam‐SS without impairing its interaction with the substrate protein.

### Suppression of Cu^II^‐dependent Protein Aggregation and Promotion of Native Folding by Cyclam‐SS

**Figure 4:**
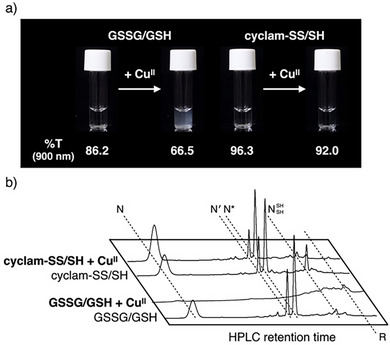
Suppression of Cu^II^‐dependent aggregation and promotion of the native folding of BPTI by cyclam‐SS. a) Effects of glutathione and cyclam‐SS on BPTI precipitation by the addition of Cu^II^ ions. The turbidity (%T) of the sample solutions at 900 nm is also shown to quantitatively compare the BPTI aggregation behaviors. b) HPLC analysis of BPTI folding reactions by glutathione and cyclam‐SS in the presence of Cu^II^ ions. [BPTI] = 30 µM, [SS compounds] = 90 µM, [SH compounds] = 900 µM, [CuCl_2_] = 0 or 180 µM. [urea] = 300 mM, 50 mM Tris–HCl (pH 7.5), 300 mM NaCl, 30 °C. The turbidity measurement: *l* = 1 cm, 30 °C. The eluent buffers for the HPLC analysis: water (containing 0.05% TFA) and CH_3_CN (containing 0.05% TFA) with a linear gradient; flow rate: 1.0 mL min^−1^; detection wavelength: 229 nm; column temperature: 50 °C.

Copper ions cause proteins to misfold and form pathological aggregates, which can lead to neurodegenerative disorders. The addition of Cu^II^ ions to R‐BPTI yielded white precipitates and increased the turbidity of the solution, indicating Cu^II^‐dependent BPTI aggregation (Figure S9). The addition of GSSG and GSH as folding promotors still resulted in the R‐BPTI solution forming a white suspension upon the addition of Cu^II^ (Figure 4a, left). In the presence of cyclam‐SS and cyclam‐SH, however, precipitation was suppressed as demonstrated by the clear R‐BPTI solution and the sustained %T value (Figure 4a, right). RP‐HPLC analysis was conducted to evaluate the folding state of BPTI in each solution (Figure 4b). HPLC profiles of the reaction mixtures after the folding of BPTI with GSSG, GSH, and Cu^II^ ions gave no peaks characteristic of N‐BPTI or folding intermediates, suggesting that BPTI had aggregated through non‐specific binding or through redox reactions of Cu^II^ ions. In contrast, the folding of BPTI was promoted by cyclam‐SS and cyclam‐SH in the presence of Cu^II^ ions, and the yield of native BPTI was improved from 26% to 42% by the addition of Cu^II^. These results indicate that cyclam‐SS has dual effects as a synthetic folding promotor: i) the suppression of Cu^II^‐dependent protein misfolding by capturing Cu^II^ ions and ii) the Cu^II^‐responsive promotion of oxidative folding by increasing its own oxidative strength in the presence of Cu^II^. BPTI folding was severely inhibited by Cu^II^ even with the mixture of GSSG/GSH/cyclam or 2‐hydroxyethyldisulfide/2‐mercaptoethanol/cyclam (Figure S10). This result supports the importance of molecular design in this study, the conjugation of cyclam ligands and an S–S bond in a single molecule, to achieve both i) suppression of metal‐dependent protein misfolding and ii) metal‐responsive promotion of proper folding.

**Figure 5:**
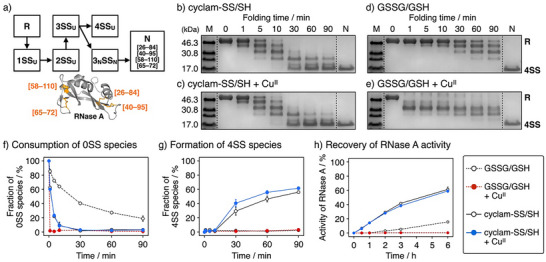
Suppression of Cu^II^‐dependent misfolding and promotion of the native folding of RNase A by cyclam‐SS. a) Folding pathway of RNase A. b)–e) Time‐course analyses of RNase A oxidation using SDS‐PAGE. f), g) Time‐course plots of the fractions of f) 0SS and g) 4SS species during RNase A folding. h) Time‐course analysis of the recovery of RNase A activity by glutathione and cyclam‐SS. The activity of RNase A was evaluated by fluorescence monitoring of the hydrolysis of oligonucleotide substrates at 30 °C. [RNase A] = 8 µM, [SS compounds] = 32 µM, [SH compounds] = 320 µM, [CuCl_2_] = 0 or 64 µM, 50 mM Tris–HCl (pH 7.5), 300 mM NaCl, 30 °C. Error bars indicate the means ± SEM of three independent experiments.

The utility of cyclam‐SS as a folding promotor under copper stress was further examined by the oxidative folding of ribonuclease A (RNase A). RNase A is a typical substrate for oxidative folding and native RNase A contains four S–S bonds: C26–C84, C40–C95, C58–C110, and C65–C72 (Figure 5a).^[^ ^61^, ^62^, ^63^ ^]^ The progress of RNase A folding from the reduced form without S─S bonds (R‐RNase A, 0SS) to the native form with four S─S bonds (N‐RNase A, 4SS) was studied by SDS‐PAGE analysis (Figure 5b–e). Since the folding efficiency was maximized by mixing cyclam‐SS and cyclam‐SH in the BPTI folding assay, we focused on the condition containing both SS and SH compounds. At each selected time point of the folding reaction, the solution was treated with malPEG‐2000 (average *M_n_* = 2000), which comprises a maleimide moiety for labeling thiol groups on RNase A with a long PEG chain to increase its apparent molecular weight. The bands for the 0SS and 4SS forms were quantified and the time‐course intensity changes were plotted as shown on Figure 5f,g, respectively. The addition of GSSG and GSH to R‐RNase A gradually consumed the 0SS form, indicating the progress of the oxidation reactions, although little 4SS species was observed in the first 90 min. The addition of Cu^II^ ions to GSSG and GSH greatly accelerated the introduction of S─S bonds, possibly due to oxidation reactions by Cu^II^ ions (Figure 5f). However, no 4SS form was observed, suggesting misfolding reactions upon mixing Cu^II^ ions with glutathione (Figure 5g). In contrast, the addition of cyclam‐SS and cyclam‐SH to the 0SS form promoted the S─S bond formation and yielded the 4SS form, along with partially oxidized intermediates. The presence of Cu^II^ ions with cyclam‐SS and cyclam‐SH further enhanced the S─S introduction to yield the 4SS form (Figure 5g). These results showed that cyclam‐SS and cyclam‐SH can suppress non‐native oxidation reactions by Cu^II^ ions and promote the introduction of native S–S bonds. Considering that the addition of cyclam to GSSG and GSH still resulted in the RNase A misfolding (Figure S11), it can be assumed that the binding of cyclam‐SS and cyclam‐SH can stabilize Cu^II^ species to prevent the unspecific oxidation by Cu^II^ and enable the stepwise oxidation by cyclam‐SS.

The folding reaction of RNase A was also studied by analyzing the recovery of enzymatic activity. Short oligonucleotides with a 6‐carboxyfluorescein (6‐FAM) and carboxytetramethylrhodamine (TAMRA) pair at each terminus and a uracil ribonucleotide as a cleavage site were used as the RNase A substrate.^[^ ^64^ ^]^ The oligonucleotide substrate was mixed with the reaction solution at each chosen time point, and the fluorescence increase caused by cleavage of the substrate was used to quantify the time‐course changes in RNase A activity (Figure 5h). The oxidative folding of RNase A by GSSG and GSH was severely limited by the addition of Cu^II^ ions, indicating that Cu^II^ ions caused RNase A misfolding and prevented the formation of native RNase A. In contrast, a mixture of cyclam‐SS and cyclam‐SH enhanced RNase A folding even in the presence of Cu^II^. In the RNase A folding experiments, Cu^II^‐responsive folding promotion was hardly observed. This is possibly because of the difference in the oxidative folding pathways, which are governed by S─S introducing reactions and S─S shuffling processes. Since RNase A has more S─S bonds (4SS) and accordingly more folding intermediates than BPTI (3SS), the S─S shuffling by cyclam‐SH was a rate‐limiting step in the RNase A folding and the Cu^II^‐dependent activation of cyclam‐SS as an oxidant was not highlighted. Thus, the SDS‐PAGE analysis and activity recovery assay show that RNase A folding by GSSG and GSH suffered from promiscuous oxidation reactions by Cu^II^ ions and resulted in the misfolding of RNase A. In contrast, cyclam‐SS and cyclam‐SH efficiently promoted the native folding of RNase A by inhibiting Cu^II^‐dependent misfolding and introducing native S─S bonds.

### Oxidative Folding of Proinsulin by Cyclam‐SS

**Figure 6:**
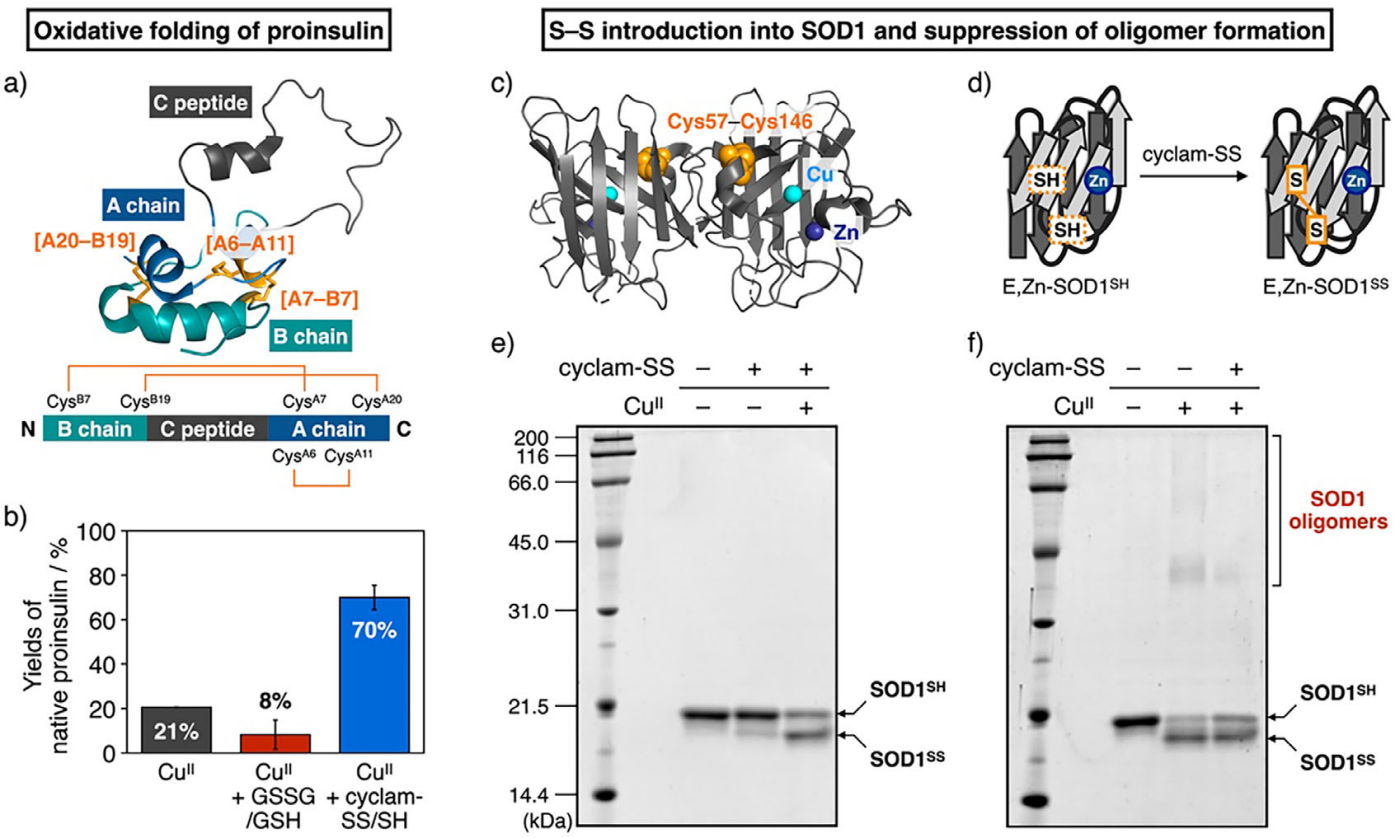
Applications of cyclam‐SS for the oxidative folding of proinsulin, the introduction of S─S bonds into reduced SOD1, and the suppression of SOD1 oligomer formation in the presence of Cu^II^ ions. a) Solution structure of proinsulin (PDB code: 2KQP). Native proinsulin contains three S─S bonds: Cys^A7^–Cys^B7^, Cys^A20^–Cys^B19^, and Cys^A6^–Cys^A11^. b) Yields of the native structure in the oxidative folding of proinsulin by SS and SH compounds in the presence of Cu^II^ ions. [proinsulin] = 5 µM, [SS compounds] = 15 µM, [SH compounds] = 150 µM, [CuCl_2_] = 30 µM, 50 mM Tris–HCl (pH 7.5), 300 mM NaCl, 30 °C. Error bars indicate the means ± SEM of three independent experiments. c) Homo‐dimeric structure of Cu,Zn‐superoxide dismutase 1 (Cu,Zn‐SOD1, PDB code: 1HL5). d) Oxidation of SOD1 containing a Zn^II^ ion and with the Cys57–Cys146 bond reduced, E,Zn‐SOD1^SH^. e) SDS‐PAGE analysis of E,Zn‐SOD1^SH^ oxidation by cyclam‐SS in the absence and in the presence of Cu^II^ ions. f) SDS‐PAGE analysis of Cu^II^‐dependent oligomerization of SOD1 with the G37R mutation in the absence and in the presence of cyclam‐SS. [SOD1] = 10 µM, [cyclam‐SS] = 0 or 10 µM, [CuCl_2_] = 0 or 10 µM, 50 mM MOPS (pH 7.0), 100 mM NaCl, 37 °C.

We further investigated the utility of cyclam‐SS as a folding promotor under copper stress by attempting the oxidative folding of aggregation‐prone proteins. We chose human proinsulin as a substrate; it is a precursor of insulin and used as a pharmaceutical peptide for treating diabetes. Human proinsulin comprises three regions, A chain, B chain, and C peptide connecting the A and B chains. Oxidative folding of human proinsulin requires the formation of two inter‐chain disulfide bonds (Cys^A7^–Cys^B7^ and Cys^A20^–Cys^B19^) and one intra‐chain disulfide (Cys^A6^–Cys^A11^) (Figure 6a). The C peptide assists the association of the two chains and promotes efficient folding, but proinsulin is prone to misfolding and aggregation due to the formation of non‐native S─S bonds.^[^ ^65^, ^66^ ^]^ Transition metal ions such as Cu^II^, Zn^II^, and Fe^II^ can also induce the aggregation of insulin at neutral pH.^[^ ^67^ ^]^ We therefore studied the utility of cyclam‐SS for the oxidative folding of proinsulin under pathological conditions, e.g., in the presence of copper ions. The progress of folding was studied by RP‐HPLC and the yields of native proinsulin were quantified using the method we previously reported (Figure 6b).^[^ ^68^ ^]^ The addition of Cu^II^ ions to reduced proinsulin provided a yield of native proinsulin of 21% after a 3 h reaction. The oxidative folding reaction partially proceeded, possibly due to the redox activity of Cu^II^ ions, but non‐specific oxidation reactions by Cu^II^ resulted in the formation of non‐native S─S bonds and misfolded proinsulin. The addition of glutathione, GSSG and GSH, did not improve the folding of proinsulin in the presence of Cu^II^ ions. In contrast, the addition of cyclam‐SS and cyclam‐SH dramatically promoted proinsulin folding to a 70% yield of the native form in the presence of Cu^II^. These results demonstrated that cyclam‐SS can efficiently promote the oxidative folding of human proinsulin by suppressing metal‐dependent misfolding and introducing native S─S bonds.

### Promotion of Native Disulfide Bonding and Suppression of Cu^II^‐dependent Oligomerization of Superoxide Dismutase 1 (SOD1)

Misfolding and aggregation of proteins can be a cause of neurodegenerative disorders. The misfolding of superoxide dismutase 1 (SOD1) is reportedly related to the pathogenesis of amyotrophic lateral sclerosis (ALS).^[^ ^69^ ^]^ Approximately 90% of ALS cases are sporadic (sALS), with unknown onset mechanisms, while the remaining 10% are familial (fALS), including about 20% of cases deriving from mutations in the SOD1 gene.^[^ ^70^, ^71^ ^]^ The fALS cases caused by the SOD1 mutation are featured by the formation of misfolded SOD1 aggregates in the spinal cords. Mature SOD1 contains an intramolecular S─S bond (Cys57–Cys146) and a metal‐binding site for a copper and zinc ion, and forms a homo‐dimeric structure (Figure 6c). The S–S bond and metal ions are largely responsible for the structural stability of SOD1, and a lack of copper and zinc ions leads to the misfolding and aggregation of SOD1.^[^ ^72^ ^]^ The suppression of SOD1 aggregation and introduction of the native S─S bond are key for maintaining the native state of SOD1. We used cyclam‐SS for the oxidation of SOD1 and to suppress its oligomerization driven by Cu^II^ ions.

We examined the oxidation of SOD1 using E,Zn‐SOD1^SH^, in which a binding site for the zinc ion is occupied by Zn^II^ and the Cys57–Cys146 bond is reduced (Figure 6d). Introduction of the Cys57–Cys146 bond was monitored by SDS‐PAGE to distinguish the reduced and oxidized forms of SOD1, E,Zn‐SOD1^SH^ and E,Zn‐SOD1^SS^, respectively (Figure 6e). The addition of 1 equiv. of cyclam‐SS to E,Zn‐SOD1^SH^ predominantly provided the E,Zn‐SOD1^SH^ band, whereas in the presence of Cu^II^ ions (1 equiv.), a different band with higher mobility appeared. This band was not observed after treatment with the reductant 2‐mercaptoethanol (Figure S12). Accordingly, it was indicated that the second band corresponds to oxidized SOD1, E,Zn‐SOD1^SS^, and that the oxidation of SOD1 to E,Zn‐SOD1^SS^ was promoted by synergistic effects of cyclam‐SS and Cu^II^. These results are consistent with the Cu^II^‐dependent activation of cyclam‐SS discussed above. Thus, we showed that cyclam‐SS can be used to promote the oxidation of SOD1, especially in the presence of Cu^II^ ions.

We then used fALS‐related mutant SOD1 as a substrate for the oxidation reaction in order to examine whether cyclam‐SS can suppress pathological SOD1 aggregation. We selected the G37R mutant, a typical pathological species in fALS that has significantly lower thermal stability than the wild type.^[^ ^73^ ^]^ In the presence of Cu^II^ ions, G37R‐mutant SOD1^SH^ was oxidized to SOD1^SS^ and oligomerized due to promiscuous oxidation reactions by Cu^II^ ions (Figure 6f). The resulting low‐mobility bands disappeared on SDS gels following treatment with 2‐mercaptoethanol, showing that these oligomers resulted from the formation of intermolecular S–S bonds (Figure S13). In contrast, in the presence of cyclam‐SS, the oxidized SOD1 oligomer bands became fainter while the oxidation of Cys57 and Cys146 was still promoted by cyclam‐SS, indicating that complexation of cyclam‐SS with Cu^II^ ions can inhibit Cu^II^‐dependent SOD1(G37R) oligomerization and promote introduction of the native S─S bond, Cys57–Cys146, thereby stabilizing the native state.

### Suppression of Cellular Copper Stress by Cyclam‐SS

**Figure 7:**
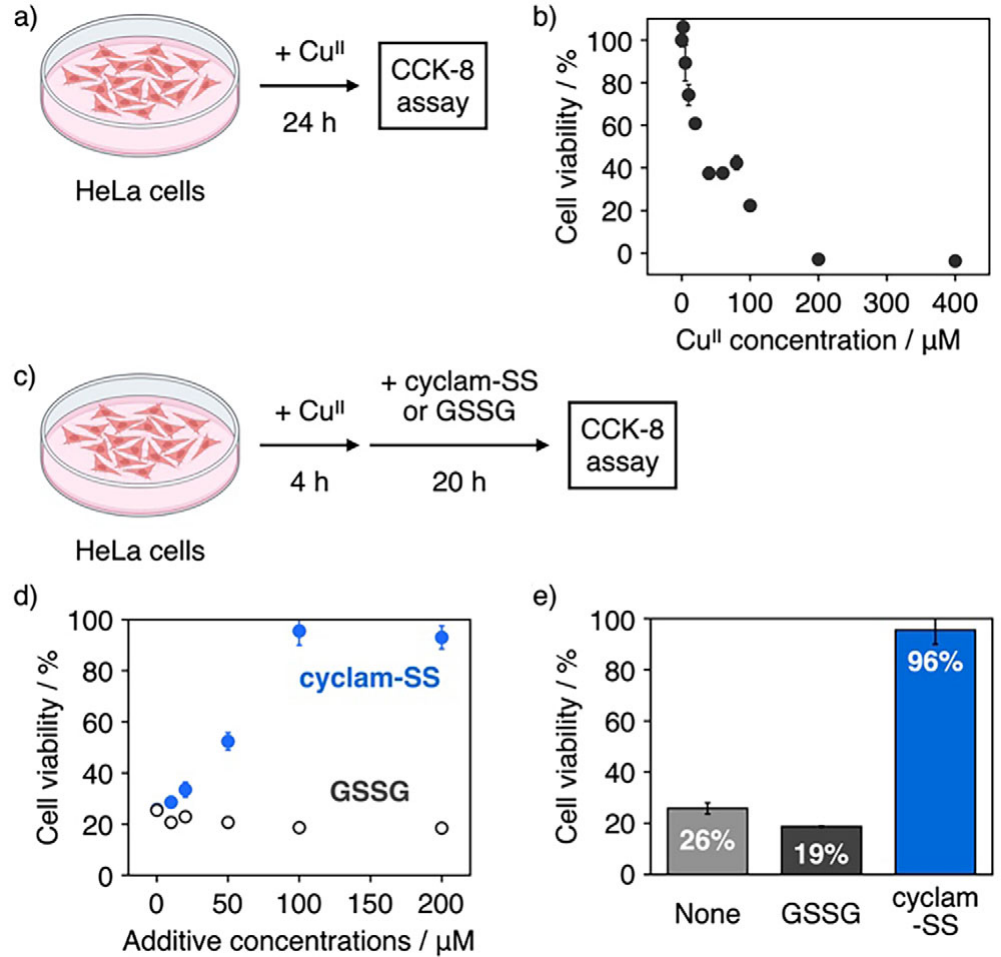
The suppression of cellular copper stress by cyclam‐SS. a) Scheme showing the cell viability assay used to evaluate Cu^II^ cytotoxicity against HeLa cells. b) Cu^II^‐dependent decrease in the viability of HeLa cells. c) Scheme showing the cell viability assay used to study the effects of cyclam‐SS and glutathione on Cu^II^ cytotoxicity. d) Effects of cyclam‐SS and glutathione on the viability of HeLa cells treated with Cu^II^ ions (100 µM). e) Viability of Cu^II^‐treated HeLa cells in the absence and in the presence of cyclam‐SS or glutathione (100 µM). ‘CCK‐8′ stands for ‘cell counting kit‐8′ assay for evaluating cell viability. Error bars indicate the means ± SEM of three independent experiments. Cell illustrations in a) and c) were created with Biorender.com.

Copper ions exhibit cytotoxicity because their binding to proteins results in protein aggregation, and their redox activity causes oxidative damage to biomolecules. Encouraged by the above‐mentioned properties of cyclam‐SS to inhibit Cu^II^‐dependent misfolding and aggregation, we examined the utility of cyclam‐SS as a synthetic reagent to suppress copper stress. Human cervical cancer (HeLa) cells are severely damaged by Cu^II^ ions^[^ ^74^ ^]^ and so we used this cell line to evaluate the effects of cyclam‐SS. The cytotoxicity of Cu^II^ ions was first studied by treating HeLa cells with various concentrations of CuCl_2_ aqueous solution and evaluating their viability using the cell counting kit‐8 (CCK‐8) assay (Figure 7a). As shown on Figure 7b, the viability of HeLa cells gradually decreased as the concentration of Cu^II^ ions increased, indicating Cu^II^‐dependent cell death. We chose the condition of 100 µM Cu^II^ ions, under which only 26% of the HeLa cells survived. The protective effects of cyclam‐SS were studied by adding cyclam‐SS to HeLa cells preincubated with Cu^II^ ion for 4 h. The preincubation time was determined according to a previous study reporting that the viability of HeLa cells is not diminished 4 h after the addition of Cu^II^ and starts decreasing after 8 h.^[^ ^74^ ^]^ As expected, the addition of cyclam ligand showed protective effects against Cu^II^ ions likely due to the sequestration of the metal ions (Figure S14). Importantly, the addition of cyclam‐SS to Cu^II^‐treated HeLa cells gradually increased cell viability in a concentration‐dependent manner and mostly protected cells from Cu^II^‐induced damaging effects at 100 µM cyclam‐SS, which is stoichiometric to the amount of Cu^II^ (Figure 7c,d). In contrast, the addition of GSSG did not improve cell viability within the concentration range tested. As shown in Figure 7e, cyclam‐SS remarkably aided the recovery of Cu^II^‐treated cell viability compared with conditions lacking additives or with GSSG. The efficacy of cyclam‐SS in Cu^II^‐induced cell death was also confirmed using live/dead cell imaging assay (Figure S15). It should be noted that the mixture of cyclam and 2‐hydroxyethyldisulfide recovered the viability of Cu^II^‐treated HeLa cells in the same manner as cyclam‐SS or cyclam (Figures S16 and S17), indicating the covalent linkage between cyclam and the disulfide linker does not spoil the function of cyclam‐SS as a Cu^II^ scavenger. These data demonstrated that cyclam‐SS can be useful for suppressing intracellular copper stress likely by maintaining native protein status in living cells. Further detailed investigations, including the functionalities of cyclam‐SS in living cells under metal‐induced stress conditions, are under way and will be reported in the future.

## Conclusion

In this study, we were inspired by natural stress response systems to develop a synthetic promotor of oxidative protein folding, cyclam‐SS, whose reactivity is up‐regulated under copper stress. Redox potential measurements revealed that the oxidative ability of cyclam‐SS is enhanced by the complexation with Cu^II^ ions. Folding experiments with BPTI showed that cyclam‐SS can promote oxidative folding more efficiently than does glutathione (GSSG). NMR analysis suggested that weak interaction between cyclam‐SS and denatured BPTI prevents the intermolecular aggregation of BPTI and promotes intramolecular interaction and disulfide formation, supporting the folding of BPTI into its native structure. Furthermore, folding reactions of BPTI and RNase A by cyclam‐SS in the presence of Cu^II^ ions highlighted bifunctional features of cyclam‐SS: i) the suppression of Cu^II^‐dependent protein misfolding and ii) the Cu^II^‐responsive promotion of the native folding process. Cyclam‐SS was then used to promote the folding of pharmaceutical and pathological proteins. Oxidative folding of proinsulin, a precursor of insulin and a typical aggregation‐prone protein, was promoted by cyclam‐SS even in the presence of Cu^II^ ions. In addition, cyclam‐SS assisted the folding of SOD1 by introducing a native S─S bond and suppressing pathological oligomerization in the presence of Cu^II^ ions. Finally, a viability assay using HeLa cells in the presence of Cu^II^ ions revealed that cyclam‐SS is functional for protecting cultured cells against copper stress.

Researchers have developed synthetic folding promotors, including redox‐active disulfide compounds, through the rational design of their chemical structures.^[^ ^38^, ^41^, ^42^, ^43^, ^44^, ^45^, ^46^, ^47^, ^48^, ^75^ ^]^ Some of the reported disulfide and thiol compounds show more efficient promotion of oxidative protein folding compared with cyclam‐SS/SH in this study (Table 2). However, their performance as folding promotors is not adaptable, whereas natural folding systems adjust to continuously changing environments by tuning their own folding capacity. For instance, although the BPTI folding by cyclam‐SS and cyclam‐SH is less efficient (53% yield at 3 h) than the recently reported pMePySS/pMePySH system (71% yield),^[^ ^49^, ^68^ ^]^ it can be up‐regulated in response to Cu^II^ ions (66% yield) up to the similar level. Furthermore, the reported systems are inherently vulnerable to Cu^II^‐dependent misfolding. In this study, we introduced metal‐binding ligands to endow the disulfide compound with stress‐responsive properties and demonstrated its applicability for aggregation‐prone and pathological proteins. The strategy of inducing the “stress‐responsive up‐regulation” process will help pave the way to the development of protein folding promotors operational under cellular stress as the next generation of synthetic chaperones and enzymes.

**Table 2:** Comparison of cyclam‐SS and cyclam‐SH with other reported disulfide and thiol reagents for promotion of oxidative protein folding.

SS compounds (conc.)	SH compounds (conc.)	Additives (conc.)	Protein (number of S–S bonds) (conc.)	Yield of N form compared with GSSG/GSH (%)	Incubation time (h)	pH	References
cyclam‐SS (90 µM)	cyclam‐SH (900 µM)	–	reduced BPTI (3SS) (30 µM)	151	1	7.5	this work
cyclam‐SS (90 µM)	cyclam‐SH (900 µM)	Cu^II^ (180 µM)	reduced BPTI (3SS) (30 µM)	247	1	7.5	this work
pMePySS (90 µM)	pMePySH (360 µM)	–	reduced BPTI (3SS) (30 µM)	209	2	7.5	[49, 68]
GSeSeG (20 µM)	–	–	reduced RNase A (4SS) (5 µM)	217	0.5	8.0	[43]
disulfide of Arg–Cys–Gly (1 mM)	Arg–Cys–Gly (2 mM)	–	reduced lysozyme (4SS) (14 µM)	130	48	8.0	[45]

Metal ions show characteristic features such as selective binding and redox activity. However, they can also cause cytotoxicity, especially when homeostasis is imbalanced. Many synthetic compounds have been designed to treat metal‐induced cellular stress, but most depend only on chelation and excretion of the metal (Table 3).^[^ ^76^, ^77^, ^78^ ^]^ For instance, Wilson's disease, which is caused by inactivation of the copper transporter ATP7B and the resulting copper overload in tissues, can normally be treated by copper‐chelating compounds such as penicillamine.^[^ ^76^ ^]^ Several cyclam derivatives have also been reported to be effective to suppress metal‐induced Aβ aggregation.^[^ ^34^, ^78^ ^]^ Cyclam‐SS, described in this study, not only utilizes the binding property of copper ions to stably capture them and mask their toxic features, but also up‐regulates itself to promote native oxidative folding. Thus, the concept behind this study is not only to detoxify copper ions, but also to utilize inherent characteristics of copper ions for molecular activation. To our knowledge, this study is the first demonstration of a synthetic bifunctional compound which both i) suppresses metal‐induced stress and ii) promotes protein folding in response to metal‐induced stress. The stress‐responsive regeneration of native protein folding status would be a new strategy for disease treatment and could be further generalized using other chelators binding to copper ions or targeting different metals. This study will contribute to the development of novel biomimetic pharmaceuticals and materials effective in preventing and treating metal‐induced stress‐related disorders including Wilson's disease, as well as enhancing the production yields of aggregation‐prone proteins in their native forms.

**Table 3:** Comparison of cyclam‐SS and cyclam‐SH with other reported chelating reagents for suppression of metal‐induced stress and diseases.

Chelating compounds	Binding metal ions	Additional functions	Target diseases	References
cyclam‐SS/SH	Cu, Ni	promotion of oxidative protein folding	–	this work
penicillamine	Cu	metal excretion in urine	Wilson's disease	[76]
dimercaptosuccinic acid	As, Hg, Pb	metal excretion in urine	heavy metal poisoning	[77]
2‐[(8‐hydroxyquinolinyl)methylene]hydrazinecarboxamide	Cu	suppression of metal–Aβ aggregation	Alzheimer's disease	[78]
cyclam and its derivatives	Cu	suppression of metal–Aβ aggregation	Alzheimer's disease	[34]

## Supporting Information

Experimental details and methods, additional figures, and characterization data of the compounds, together with additional references^[^ ^79^, ^80^, ^81^, ^82^, ^83^ ^]^ can be found in the Supporting Information.

## Conflict of Interests

The authors declare no conflict of interest.

## Supporting information



Supporting Information

## Data Availability

The data that support the findings of this study are available in the Supporting Information of this article
