# Supplementary material for: Metal‐Responsive Up‐Regulation of Bifunctional Disulfides for Suppressing Protein Misfolding and Promoting Oxidative Folding
Source: Angew Chem Int Ed Engl. 2025 Jun 30;64(36):e202502187. doi: 10.1002/anie.202502187 (PMC12402870; doi:10.1002/anie.202502187)
Supplement: Supplementary file 1 — Supporting Information [file ANIE-64-e202502187-s001.pdf]

Supporting Information

Metal-Responsive Up-regulation of Bifunctional Disulfides for Suppressing Protein Misfolding and Promoting Oxidative Folding

Keita Mori,*[a,b] Tsubura Kuramochi,[c] Motonori Matsusaki,[d] Yuki Hashiguchi,[e] Masaki Okumura,[c] Tomohide Saio,[d] Yoshiaki Furukawa,[e] Kenta Arai,[f] Takahiro Muraoka*[a,g]

[a] Department of Applied Chemistry, Graduate School of Engineering, Tokyo University of Agriculture and Technology, 2-24-16 Naka-cho, Koganei, Tokyo 184-8588, Japan.

[b] Department of Chemistry, Massachusetts Institute of Technology, 77 Massachusetts Avenue, Cambridge, MA 02139, USA.

[c] Frontier Research Institute for Interdisciplinary Sciences, Tohoku University, 6-3 Aramaki-Aza-Aoba, Aoba-ku, Sendai, Miyagi 980-8578, Japan.

[d] Institute of Advanced Medical Sciences, Tokushima University, 3-18-5 Kuramoto-cho, Tokushima 770-8503, Japan.

[e] Department of Chemistry, Keio University, 3-14-1 Hiyoshi, Kohoku, Yokohama, Kanagawa 223-8522, Japan.

[f] Department of Chemistry, School of Science, Tokai University, 4-4-1 Kitakaname, Hiratsuka, Kanagawa 259-1292, Japan.

[g] Kanagawa Institute of Industrial Science and Technology, 3-2-1 Sakato, Takatsu-ku, Kawasaki, Kanagawa 213-0012, Japan.

Contents

1. Materials ····S3

2. Instrumentation ····S3

3. Methods ····S4

4. Synthesis ····S8

5. Supplementary Tables and Figures ···S10

6. References ···S16

1. Materials

p-Toluenesulfonyl chloride, copper(II) chloride dihydrate, nickel(II) chloride hexahydrate, zinc(II) sulfonate hexahydrate, hydrochloric acid, and maleic acid were purchased from FUJIFILM Wako Chemicals (Osaka, Japan). Deuterium oxide (D_2O), dry acetonitrile, potassium carbonate, and di-*tert*-dicarbonate were purchased from Kanto Chemical (Tokyo, Japan). Sodium hydroxide (NaOH) and trifluoroacetic acid (TFA) were purchased from Kishida Chemical (Tokyo, Japan). Coomassie brilliant blue G-250, 5,5′-dithiobis(2-nitrobenzoic acid) (DTNB), 1,4-dithiothreitol (DTT), L-glutathione oxidized (GSSG), L-glutathione reduced (GSH), guanidine hydrochloride (GdnHCl), 6 M hydrochloric acid, urea, di-sodium hydrogenphosphate 12-water, sodium dihydrogenphosphate dihydrate, ammonium sulfate, sodium acetate trihydrate, di-sodium dihydrogen ethylenediamine tetraacetate dihydrate (EDTA·2Na·$2H_2O$), 3-morpholinopropanesulfonic acid (MOPS), iodoacetamide, and 2-mercaptoethanol were purchased from Nacalai Tesque (Kyoto, Japan). Bovine pancreatic trypsin inhibitor (BPTI) was purchased from Pro-Spec-Tany TechnoGene (Rehovot, Israel). 2-Hydroxyethyl disulfide, triethylamine, maleimidePEG-2000 (malPEG-2000), ribonuclease A (RNase A) from bovine pancreas, and tris(2-carboxyethyl)phosphine (TCEP) hydrochloride solution were purchased from Sigma-Aldrich (St. Louis, MO, USA). 1,4,8,11-Tetraazacyclotetradecane and tris(3-hydroxypropyltriazolylmethyl)amine were purchased from Tokyo Chemical Industry (Tokyo, Japan). An oligonucleotide substrate for the RNase A refolding assay (sequence: 5′-FAM-dA rU dA dA-TAMRA-3′) was purchased from Japan Bio Services after purified by HPLC and used without purification. Cell counting kit-8 was purchased from Dojindo (Kumamoto, Japan). A live/dead cell imaging kit was purchased from Invitrogen (Waltham, MA, USA). Deionized water (filtered through a 0.22 μm membrane filter, >18.2 MΩ cm) was purified in Purelab DV35 of ELGA (Buckinghamshire, UK) and a Milli-Q system of Merck Millipore (Burlington, MA, USA). Column chromatography was carried out with Silica Gel 60 (spherical, neutral, particle size: 63–210 μm) purchased from Kanto Chemical (Tokyo, Japan). Glass-backed silica gel was purchased from Merck (Darmstadt, Germany).

2. Instrumentation

Nuclear magnetic resonance (NMR) spectra were recorded on JNM-ECX 400 spectrometer (400 MHz for ^{1}H) or JNM-ECA 500 spectrometer (500 MHz for ^{1}H) of JEOL (Tokyo, Japan). The spectra were referenced to tetramethylsilane (TMS) in $CDCl_3$ (δ 0 ppm), 3-(trimethylsilyl)propionic-*2*,*2*,*3*,*3*-d_4 acid in D_2O (δ 0 ppm for ^{13}C), or the residual solvent signal in D_2O (δ 4.65 ppm for ^{1}H). NMR spectra for ^{15}N BPTI were recorded on Bruker AVANCE III 500 MHz NMR instrument equipped with a BBO probe. High-resolution matrix-assisted laser desorption/ionization time-of-flight mass (MALDI-TOF MS) spectra were recorded on autoflex speed of Bruker. High-resolution electrospray ionization time-of-flight mass (HR ESI TOF MS) spectra were recorded on a Bruker micrOTOF-QII.

UV-vis absorption spectra were recorded on V-750 UV-Vis spectrophotometer of JASCO (Tokyo, Japan). Time-course fluorescence analysis was conducted with Shimadzu type RF-6000 spectrofluorometer. Analytical reversed-phase high-performance liquid chromatography (RP-HPLC) was conducted with Primaide HPLC

system of HITACHI (Tokyo, Japan) using TSKgel Protein C4-300 column of Tosoh Bioscience (φ4.6 × 150 mm, Tokyo, Japan) for BPTI folding assay and JASCO HPLC system using YMC Triart C18 column (φ4.6 × 250 mm, Kyoto, Japan) for redox potential measurements and TSKgel ODS-100V 5 µm column (Tosoh Bioscience, Japan) for proinsulin folding assay. Semi-preparative reversed-phase high-performance liquid chromatography was performed on PU-4086-Binary pump, UV-4075 detector and CHF122SC fraction collector of JASCO (Tokyo, Japan) attached with TA12S05-2520WX Actus Triart column of YMC (φ20 × 250 mm, Tokyo, Japan). Fluorescent microscopic observation was performed with an Olympus IX-73 microscope.

3. Methods

1) Determination of thiol concentration for oxidative protein folding assay: A thiol compound dissolved in 10 mM HCl aq. was diluted in a buffer (50 mM Tris-HCl, 0.3 M NaCl, pH 7.5). The mixture was added to an aqueous solution of 5,5'-dithiobis(2-nitrobenzoic acid) (DTNB) and incubated for 10 min at 30 °C. The concentration of the thiol compound was determined by the absorbance at 412 nm measured at 30 °C with V-750 UV-Vis spectrophotometer.[1]

2) Redox potential $E°'$ measurements: $E°'$ values of the thiol compounds were determined by following the protocol described in a previous paper.[2] A buffer (100 mM Tris-HCl, pH 7.0) was degassed with N_2 for 30 min prior to use. Reduced DTT (DTT^{red}, 60 µM, 2.4 mL) in the buffer was added to a disulfide compound (60 µM, 2.4 mL) in the buffer and the resultant mixture was incubated under N_2 at 25 ± 0.1 °C for 24 h. To quench the reaction, an aliquot of the reaction mixture (1.0 mL) was added to 1 M HCl aq. (200 µL), and the obtained solution was immediately analyzed by RP-HPLC (YMC Triart C18 column, φ4.6 × 250 mm). The RP-HPLC analysis was conducted with water/acetonitrile = 98/2 containing 0.1% TFA. The concentrations of the species at equilibrium were calculated from the observed peak areas and the corresponding calibration curves.
The equilibrium constant K_{eq} for the reaction (eq. 2), described as eq. 3, was determined by averaging three times of individual experiments following the above procedure.

$$\text{Disulfide} + \text{DTT}^{red} \rightleftarrows 2\text{Thiol} + \text{DTT}^{ox} \quad (2)$$

$$K_{eq} = \frac{[\text{Thiol}]^2[\text{DTT}^{ox}]}{[\text{Disulfide}][\text{DTT}^{red}]} \quad (3)$$

The redox potential $E°'$ was calculated by the Nernst's equation (eq. 4)

$$E°' = E°'_{DTT} + \frac{RT}{nF} \cdot \ln K_{eq} \qquad (4)$$

where n is the number of transferred electrons (n = 2), F is Faraday's constant (96500 C mol^{-1}), R is the universal gas constant (8.314 J K^{-1} mol^{-1}), T is the temperature (298 K), and $E°'_{DTT}$ is the redox potential of DTT (–327 mV).

3) Determination of cyclam-SH pK_a value: Stock solutions of acetate buffer (CH_3COOH and CH_3COONa for pH 3.0–5.5), phosphate buffer (Na_2HPO_4 and KH_2PO_4 for pH 5.5–8.0), borate buffers ($Na_2B_4O_7$ and HCl for

pH 8.0–9.0, $Na_2B_4O_7$ and NaOH for pH 9.0–11.0) were degassed with N_2 for 1 h immediately prior to use. A stock solution of cyclam-SH in degassed water (4.0 mM) was then prepared. Immediately after the aqueous solution of cyclam-SH (5 μL) and a buffer (395 μL) were combined in a 1-cm thick quartz cuvette, the UV absorption spectrum of the sample was measured. The pH value of the sample was measured by a HORIBA pH meter (9618S-10D), which had been calibrated prior to use with pH 4.01, 6.86 and 9.18 standard solutions (HORIBA 101-S). Absorbance values at 240 nm of the samples were plotted in the function of the pH values, and the pK_a value of the thiol compound was calculated with KaleidaGraph software (version 5.0.3) by a curve fitting analysis using the following equation:

$$y = a + (m1)/(m2*10^{\wedge}(-x) + 1);\ m1 = b;\ m2 = 1000 \qquad (5)$$

where a is the absorbance of a sample below pH 4 and b is the difference of absorbances above the pH 10 and below the pH 4, and curve fitting calculation provides m2 = pK_a. The r^2 value was higher than 0.99.

4) Evaluation of nucleophilicity of thiol compounds: A solution of DTNB (5 μM) in a buffer (50 mM Tris–HCl, 300 mM NaCl, pH 7.5) was prepared and incubated at 30 °C. Stock solution of the thiol compounds was added and absorbance change at 412 nm was recorded on 750 UV-Vis spectrophotometer at 30 °C. For the sample with cyclam-SH and Cu^{II}, cyclam-SH and Cu^{II} were mixed and preincubated for 10 min at 30 °C before mixed with DTNB solution.

5) BPTI folding assay: BPTI (10 mg) dissolved in 0.1 M Tris (pH 8.0, 1.0 mL) containing 30 mM DTT and 8 M urea was incubated for 3 h at 50 °C. Fully reduced and denatured BPTI was purified by RP-HPLC using an InertSustain C18 column (φ4.6 × 250 mm, GL Sciences, Tokyo Japan), and the collected fractions were lyophilized. The obtained powder was stored at –30 °C until use. For the assay, a disulfide compound was dissolved in a buffer (50 mM Tris-HCl, 300 mM NaCl, pH 7.5) with or without a thiol compound and metal ions, and the resulting mixture was preincubated for 10 min at 30 °C. To the preincubated mixture, fully reduced and denatured BPTI (600 μM) dissolved in 6 M urea containing 0.05% trifluoroacetic acid was added (final concentration of BPTI: 30 μM, urea: 300 mM, disulfide compound: 90 μM, thiol compound: 900 μM, Tris-HCl: 50 mM, NaCl: 300 mM, pH 7.5). After the incubation at 30 °C for predetermined periods, the reaction was quenched by adding an equal volume of 1 M HCl aq., which was then analyzed by RP-HPLC at a flow rate of 1.0 mL min^{-1} monitoring at 229 nm with a linear gradient of elutions (solvent A: 0.05% trifluoroacetic acid in water; solvent B: 0.05% trifluoroacetic acid in acetonitrile; percentages of solvent A: 95% at 0 min, 80% at 15 min, 65% at 45 min).

6) Expression and purification of the recombinant BPTI All-Ser: cDNA encoding BPTI were subcloned into the NdeI and EcoRI sites of the pET17b vector (Novagen, Germany). BPTI mutant in which all the cysteine residues were replaced with serine residues (BPTI All-Ser) were constructed by using a PrimeSTAR Mutagenesis Basal Kit (Takara Bio, Japan). BPTI All-Ser was overexpressed in *Escherichia coli* strain BL21(DE3) by culturing at 37°C overnight. The cells were disrupted in the buffer containing 50 mM Tris-HCl (pH 8.1), 300 mM NaCl, and 1 mM phenylmethylsulfonyl fluoride using a homogenizer (Sonics and Materials,

USA). By the centrifugation of the homogenized lysate, recombinant BPTI all-Ser was obtained as an inclusion body. The inclusion body was dissolved in 100 mM Tris-HCl (pH 8.0) containing 8 M urea and 20 mM dithiothreitol at 50 °C for 1 h. After centrifugation, the supernatant was loaded onto a COSMOSIL 5C18 column (Nacalai Tesque, Japan), and BPTI All-Ser was eluted with the 80% CH_3CN in 0.05% TFA. The eluted samples were finally purified by the RP-HPLC (GL Science, Japan) equipped with a COSMOSIL 5C18-AR-II column (Nacalai Tesque, Japan) using a linear gradient of CH_3CN in 0.05% TFA. Purified samples were lyophilized.

7) NMR interaction study for BPTI All-Ser: ^{15}N-labeled BPTI All-Ser were prepared at 100 μM concentration in 50 mM HEPES-NaOH pH 7.0, 10% D_2O, and subjected to NMR measurement on Bruker Avance III 500 MHz NMR spectrometer equipped with BBO probe with sample temperature of 10 or 30 °C. ^{1}H–^{15}N selective optimized flip angle short transient (SOFAST)-heteronuclear multiple quantum correlation (HMQC) spectra[3] of ^{15}N-labeled BPTI All-Ser were acquired in the absence and presence of cyclam-SS (0, 1, or 5 mM), GSSG (0 or 1 mM) and Cu^{II} ions (0, 1, or 5 mM). The spectra were processed using NMRPipe program[4] and analyzed using Olivia program (https://github.com/yokochi47/Olivia). Amide resonance assignments for BPTI All- Ser were obtained with the reference of the previous study.[5]

8) Preparation of reduced and denatured RNase A: RNase A (32 mg mL^{-1}) was dissolved in a buffer (500 μL, 200 mM Tris-HCl, pH 8.7) containing 6.0 M GdnHCl and 100 mM DTT and the mixture was incubated for 2 h at 25 °C. The resulting mixture was dialyzed three times for 2 h each with 10 mM HCl aq. (1 L) to remove the denaturing and reducing reagents. After the dialysis, the sample was stored at –30 °C until use.

9) Gel shift assay of RNase A folding: Oxidative folding of RNase A (8 μM) was carried out in a buffer (50 mM Tris-HCl, 300 mM NaCl, pH 7.5) containing 32 μM disulfide compound with 320 μM thiol compound. At 0, 1, 5, 10, 30, 60, and 90 min after starting the incubation, free thiols were inactivated by the addition of Laemmli's 4×SDS-loading buffer[6] containing 10 mM malPEG-2000. RNase A was separated depending on the number of disulfide bonds by non-reducing 14% SDS-PAGE using WIDE RANGE gel (Nacalai Tesque, Kyoto, Japan). Proteins were visualized by coomassie brilliant blue G-250 staining. The gel image was imported with a ChemiDoc Touch Imaging System and the band intensities were analyzed by Image Lab software (Bio-Rad, Hercules, CA, USA).

10) RNase A refolding assay: Prior to the assay, a mixture of a disulfide compound and a thiol compound was dissolved in a buffer (50 mM Tris-HCl, 300 mM NaCl, pH 7.5) in the absence or in the presence of Cu^{II} ions, and the resulting mixture was preincubated for 5 min at 30 °C. To the preincubated mixture, fully reduced and denatured RNase A (8 μM) was added and incubated at 30 °C in a buffer (50 mM Tris-HCl, 300 mM NaCl, pH 7.5) containing 32 μM disulfide compound with 320 μM thiol compound. At 30, 60, 120, 180, and 360 min after starting the incubation, aliquots (5 μL each) were taken from the reaction solution and were immediately added to a buffer (395 μL, 50 mM Tris-HCl, 300 mM NaCl, pH 7.5). 5 μL of the obtained solution was further diluted in the buffer (495 μL) containing a substrate oligonucleotide (sequence: 5′-FAM-dA rU dA dA-TAMRA-3′,

final concentration of the substrate = 200 nM) followed by the measurement of the linear increase in fluorescence at 520 nm (λ_{ex} = 495 nm) at 30 °C. Values represent means ± SEM based on the three independent experiments.

11) Proinsulin folding assay: Reduced and denatured proinsulin (5 μM) was incubated with 30 μM $CuCl_2$ in 50 mM Tris-HCl (pH 7.5) containing 300 mM NaCl, 150 μM reductant (GSH or cyclam-SH) and 15 μM oxidant (GSSG or cyclam-SS) at 30°C. The reaction was quenched with an equivalent volume of 2-aminoethyl methanethiosulfonate (AEMTS) (7 mg mL^{-1}) at 3 h to quench their oxidative folding. Reaction mixtures were analyzed by RP-HPLC on a TSKgel ODS-100V 5 μm column (Tosoh Bioscience, Japan) with monitoring at 220 nm. The identities of the resulting peaks were confirmed by MALDI-TOF/MS analysis as described previously[7,8,9].

12) Expression and purification of recombinant SOD1: The cDNA coding SOD1 was inserted between the NcoI and SalI site of a modified pET-15b plasmid with the SalI site (Novagen). The G37R mutation was introduced by an inverse PCR method using PrimeSTAR Max DNA Polymerase (TAKARA).
SOD1 proteins without any tags were expressed in *E. coli* SHuffle™ (New England Biolabs) by culturing them with 0.5 mM isopropyl β-D-1-thiogalactopyranoside at 20 °C, 135 rpm overnight. The cell lysate was centrifuged at 20,000 x *g*, and ammonium sulfate was added to the resultant supernatant to 50% of its saturation. After centrifugation at 20,000 x *g*, the supernatant was loaded on a HiTrap Phenyl FF (high sub) column (Cytiva), and the solution eluted by a buffer containing 100 mM sodium phosphate and 100 mM NaCl at pH 7.0 with 1 M ammonium sulfate was collected. To prepare a metal-free (apo) form of SOD1, the solution was dialyzed against a buffer containing 50 mM sodium acetate, 100 mM NaCl, and 10 mM ethylenediaminetetraacetic acid (EDTA) at pH 4.0. After precipitates were removed by centrifugation at 20,000 x *g*, the supernatant was dialyzed against a buffer containing 10 mM tris(hydroxymethyl)aminomethane (Tris) and 1 mM EDTA at pH 8.0 and then loaded on a HiTrap Q FF column (Cytiva). The proteins eluted by a buffer containing 10 mM Tris, 90 mM NaCl, and 1 mM EDTA at pH 8.0 were collected, and the buffer was exchanged to 50 mM 3-morpholinopropanesulfonic acid (MOPS)/100 mM NaCl, pH 7.0 with a PD-10 column (Cytiva). SOD1 was further purified as an apo form by size-exclusion chromatography using a gel filtration column (Cosmosil 5Diol-300-II, nacalai tesque) equilibrated with 50 mM MOPS/100 mM NaCl, pH 7.0. Almost no copper and zinc ions in the purified SOD1 samples (less than 1% of SOD1) were confirmed by using graphite furnace atomic absorption spectroscopy (AA-7000, Shimadzu).
The disulfide-reduced form of SOD1 was prepared by incubation of 100 μM SOD1 in the apo form with 5 mM tris(2-carboxyethyl)phosphine) (TCEP) in 50 mM MOPS/100 mM NaCl, pH 7.0 at 50 °C for an hour. TCEP was then removed by the buffer-exchange with 50 mM MOPS/100 mM NaCl, pH 7.0, using Amicon® Ultra Centrifugal Filter, 10 kDa MWCO (Merck). The zinc-bound form of the disulfide-reduced SOD1 (E,Zn-$SOD1^{SH}$) was prepared by incubation of 20 μM disulfide-reduced SOD1 in the apo form with 20 μM $ZnSO_4$ at 37 °C for 30 min. The concentration of SOD1 was determined spectroscopically by using 5,625 cm^{-1} M^{-1} (for SOD1 with the disulfide) and 5,500 cm^{-1} M^{-1} (for disulfide-reduced SOD1) of the molar extinction coefficient.

13) SOD1 folding assay: E,Zn-SOD1^{SH} in the concentration of 10 µM was mixed with equimolar amounts of either cyclam-SS, $CuCl_2$, or both in 50 mM MOPS/100 mM NaCl, pH 7.0, and then incubated at 37 °C for 30 min. For the SDS-PAGE analysis under non-reducing conditions, the samples containing 1.2 µg of SOD1 proteins were mixed with a loading buffer (50 mM Tris, pH 6.8/1.6% SDS/10% glycerol/0.02% bromophenol blue) containing 120 mM iodoacetamide and incubated at 37 °C for 40 min. For the analysis under reducing conditions, the samples containing 1.2 µg of SOD1 proteins were mixed with the loading buffer containing 6.7% β-mercaptoethanol. In both conditions, the samples were boiled at 100 °C for 5 min, loaded on a 15% polyacrylamide gel and then electrophoresed at 150 V. Gels were stained with Coomassie Brilliant Blue R-250 to visualize protein bands.

14) Cell viability assay: Human cervix carcinoma epithelial HeLa cells were seeded in 96-well plates as 5000 cells per well in Minimum Essential Medium (MEM) with 10% fetal bovine serum (FBS) and cultured at 37 °C under 5% CO_2 for 24 hours. Copper exposure was achieved by the addition of $CuCl_2$ aqueous solution into the culture medium to obtain the desired final concentration of Cu^{II}. After 24-hour incubation at 37 °C under 5% CO_2, the cells were incubated with cell counting kit-8 (CCK-8) reagent (10 µL) for 2 hours and the absorption at 450 nm was recorded by absorption spectroscopy.
To test the protective effects of cyclam-SS, 4 hours after the addition of $CuCl_2$, Cu^{II}-treated HeLa cells were mixed with aqueous solution of cyclam-SS, GSSG, or cyclam–2-hydroxyethyldisulfide mixture (2:1). After 20-hour incubation, the cell viability was evaluated by the CCK-8 assay.
For the fluorescent imaging analysis, after the copper exposure and the incubation with the reagents, the live/dead staining solution (100 µL) was added instead of CCK-8. After 15-min incubation at room temperature, the cell viability was analyzed by fluorescent microscopy.

4. Synthesis

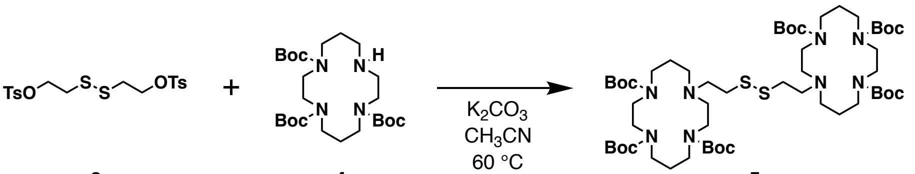

3 4 5

1) Synthesis of 5. Compounds **3** and **4** were synthesized following the reported procedures.[10,11] To an acetonitrile (5.9 mL) solution of **3** (270 mg, 0.585 mmol), K_2CO_3 and **4** (612 mg, 1.22 mmol, 2.1 eq) were added. After stirring for 5 days at 60 °C, the obtained suspension was filtered, and the filtrate was evaporated to dryness under reduced pressure. The crude product was further purified by silica gel chromatography, and compound **5** was obtained as a white foam (406 mg, 0.363 mmol, 62%).
^{1}H NMR (400 MHz, $CDCl_3$, 323 K): δ = 3.45–3.21 (m, 28H), 2.97–2.58 (m, 10H), 2.47–2.41 (m, 2H), 1.88–1.62 (m, 8H), 1.46–1.45 (m, 54H) ppm; ^{13}C NMR (125 MHz, $CDCl_3$, 323 K): δ = 155.9, 155.6, 155.4, 79.5, 79.4, 54.5, 54.2, 53.6, 51.7, 48.6, 48.2, 47.9, 47.7, 47.2, 46.9, 46.7, 46.3, 45.7, 44.8, 28.8, 28.4, 27.5, 26.8, 25.5 ppm; HR ESI TOF MS (positive mode): m/z = 1119.7132 (calculated for $C_{54}H_{103}N_8O_{12}S_2$: $[M + H]^+$ = 1119.7131).

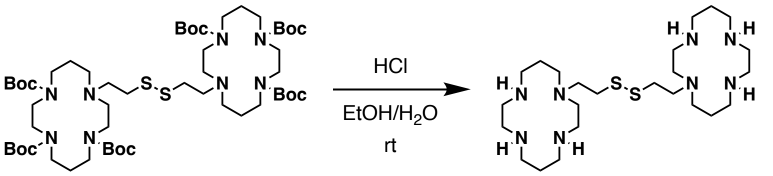

5 6

2) Synthesis of 6 (cyclam-SS). To an ethanol (520 μL) solution of **5** (112 mg, 0.100 mmol), 35% HCl aq (520 μL) was added and the solution was stirred on ice for 10 min and at room temperature for 3 h. The obtained white suspension was filtered, and a crude product was obtained as a white solid (55.2 mg). Subsequent purification by HPLC and freeze-drying gave compound **6** as a colorless film (12.4 mg, 0.0240 mmol, 24%). The yield was confirmed by NMR measurement with maleic acid as an internal standard as well.

^{1}H NMR (400 MHz, $CDCl_3$, 323 K): δ = 3.13–3.03 (m, 16H), 2.84–2.78 (m, 16H), 2.67–2.65 (m, 8H), 1.82–1.71 (m, 8H) ppm; ^{13}C NMR (125 MHz, $CDCl_3$, 323 K): δ = 54.69, 51.53, 49.34, 49.28, 45.50, 45.16, 42.54, 41.89, 33.36, 30.19, 22.51, 22.30 ppm; HR ESI TOF MS (positive mode): *m*/*z* = 260.2027 (calculated for $C_{24}H_{56}N_8S_2$: $[M + 2H]^{2+}$ = 260.2029).

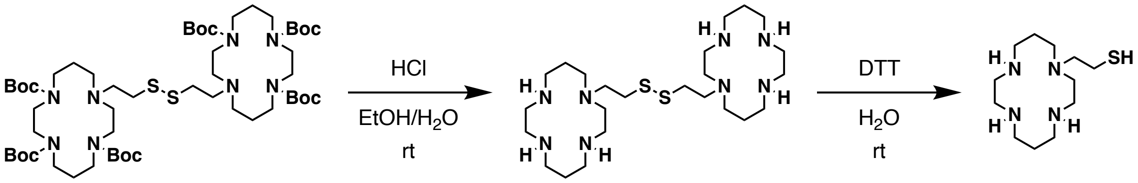

5 6 7

3) Synthesis of 7 (cyclam-SH). To an ethanol (520 μL) solution of **5** (112 mg, 0.100 mmol), 35% HCl aq (520 μL) was added and the solution was stirred on ice for 10 min and at room temperature for 3 h. The white suspension was filtered, and a crude product of **6** was obtained as a white solid (55.2 mg). The crude product was dissolved in 3 mL water and mixed with dithiothreitol (113 mg, 0.731 mmol, 7.3 eq) followed by stirring for 38 h at room temperature. The obtained solution was purified by HPLC, and the subsequent freeze-drying gave compound **7** as a colorless film (9.8 mg, 0.038 mmol, 19% in 2 steps).

^{1}H NMR (400 MHz, $CDCl_3$, 323 K): δ = 3.13–3.03 (m, 8H), 2.85–2.77 (m, 4H), 2.69–2.59 (m, 8H), 1.85–1.71 (m, 4H) ppm; ^{13}C NMR (125 MHz, $CDCl_3$, 323 K): δ = 59.32, 51.56, 48.88, 45.58, 44.95, 42.16, 41.73, 22.28, 21.62, 21.27 ppm; HR ESI TOF MS (positive mode): *m*/*z* = 261.2108 (calculated for $C_{12}H_{29}N_4S$: $[M + H]^{+}$ = 261.2107).

5. Supplementary Tables and Figures

Table S1 Comparison of redox potential between cyclam-SS and 2-hydroxyethyldisulfide.

Compound	Redox potential ($E°'$) / mV [a]
cyclam-SS	–231 ± 0.8
2-hydroxyethyldisulfide	–253 ± 0.003 [b]
2-hydroxyethyldisulfide + cyclam (1:2 mixture)	–250 ± 8.8

[a] Error values indicate the means ± SEM of three independent experiments. [b] Ref. 12.

Table S2 Comparison of the BPTI folding efficiency of cyclam-SS and 2-hydroxyethyldisulfide.

Compound	Yields of native BPTI at 1 h [a]
cyclam-SS	14.0 ± 0.3%
2-hydroxyethyldisulfide	3.8 ± 0.01%
2-hydroxyethyldisulfide + cyclam (1:2 mixture)	3.8 ± 0.2%

[a] Error values indicate the means ± SEM of three independent experiments.

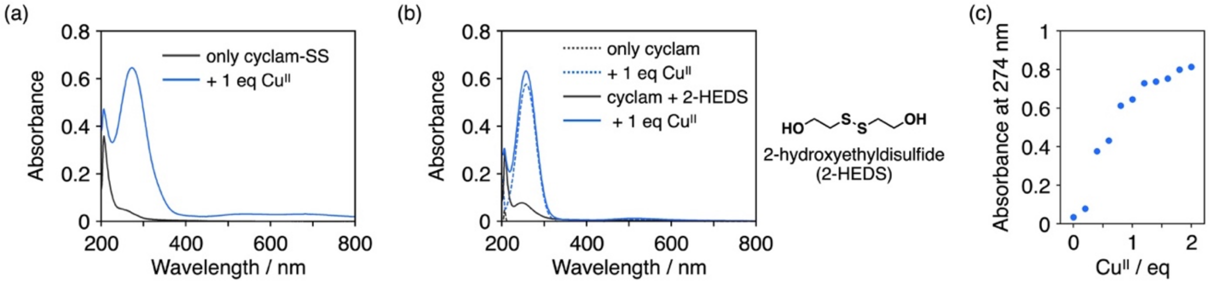

Fig. S1 UV absorption changes of cyclam-SS and cyclam derivatives in the presence of Cu^{II} ions. (a) UV–Vis absorption spectra of cyclam-SS (90 µM) in the absence and in the presence of Cu^{II} ions (90 µM). (b) UV–Vis absorption spectra of cyclam (90 µM) and the mixture of cyclam (90 µM) and 2-hydroxyethyldisulfide (2-HEDS, 45 µM) in the absence and in the presence of Cu^{II} ions (90 µM). (c) Cu^{II}-dependent changes in the absorbance of cyclam-SS at 274 nm. [cyclam-SS] = 90 µM, [$CuCl_2$] = 0–180 µM. 50 mM Tris–HCl (pH 7.5), 300 mM NaCl, l = 1 cm, 30 °C.

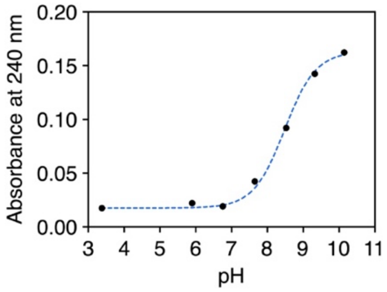

Fig. S2 Absorbance changes at 240 nm and curve fitting analyses of cyclam-SH at variable pH between 3 and 11 to evaluate the pK_a value of cyclam-SH. [cyclam-SH] = 50 µM, 30 °C.

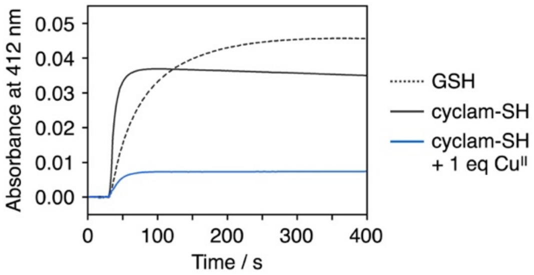

Fig. S3 Disulfide exchanging reactions of thiol compounds with DTNB monitored by absorbance changes at 412 nm. [SH compounds] = 2.5 μM, [DTNB] = 5 μM. 50 mM Tris–HCl (pH 7.5), 300 mM NaCl. l = 1 cm, 30 °C.

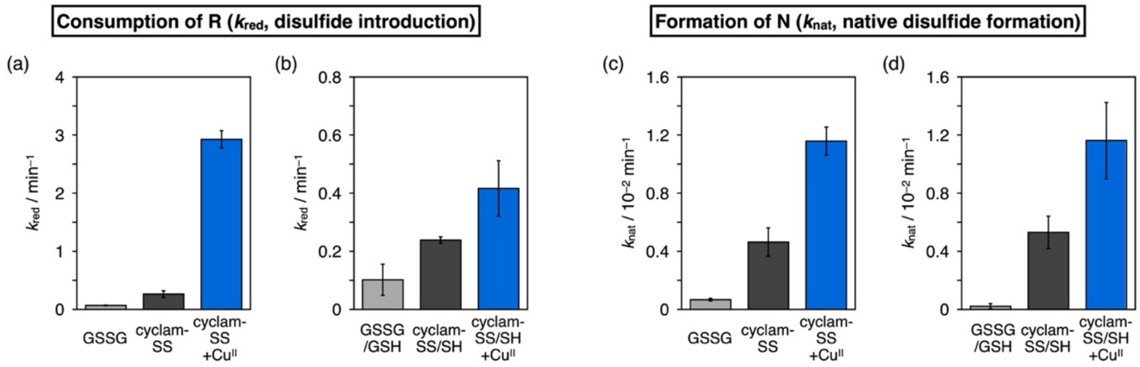

Fig. S4 Folding kinetic rates of (a,b) the disulfide introduction (k_{red}) and (c,d) the native disulfide formation (k_{nat}) during BPTI folding. [BPTI] = 30 μM, [SS compounds] = 90 μM, [SH compounds] = 0 or 900 μM, [$CuCl_2$] = 0, 90, or 180 μM. [urea] = 300 mM, 50 mM Tris–HCl (pH 7.5), 300 mM NaCl, 30 °C. Data were analyzed by curve fitting with the statistical software IGOR Pro 6.0 (WaveMetrics).

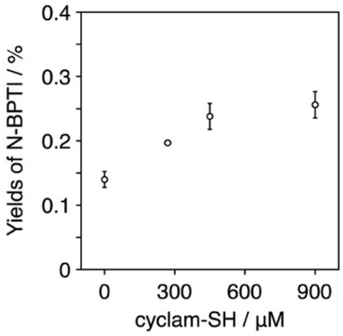

Fig. S5 Titration of cyclam-SH in the oxidative folding of BPTI by cyclam-SS. [BPTI] = 30 μM, [cyclam-SS] = 90 μM, [cyclam-SH] = 0, 270, 450, or 900 μM, [urea] = 300 mM, 50 mM Tris–HCl (pH 7.5), 300 mM NaCl, 30 °C. Error bars indicate the means ± SEM of three independent experiments.

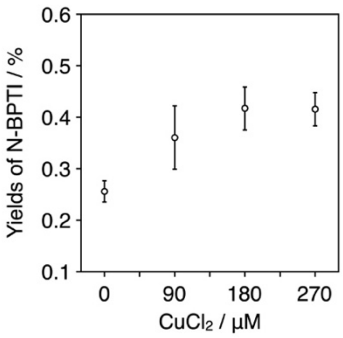

Fig. S6 Titration of $CuCl_2$ in the oxidative folding of BPTI by cyclam-SS and cyclam-SH. [BPTI] = 30 μM, [cyclam-SS] = 90 μM, [cyclam-SH] = 900 μM, [$CuCl_2$] = 0, 90, 180, or 270 μM. [urea] = 300 mM, 50 mM Tris–HCl (pH 7.5), 300 mM NaCl, 30 °C. Error bars indicate the means ± SEM of three independent experiments.

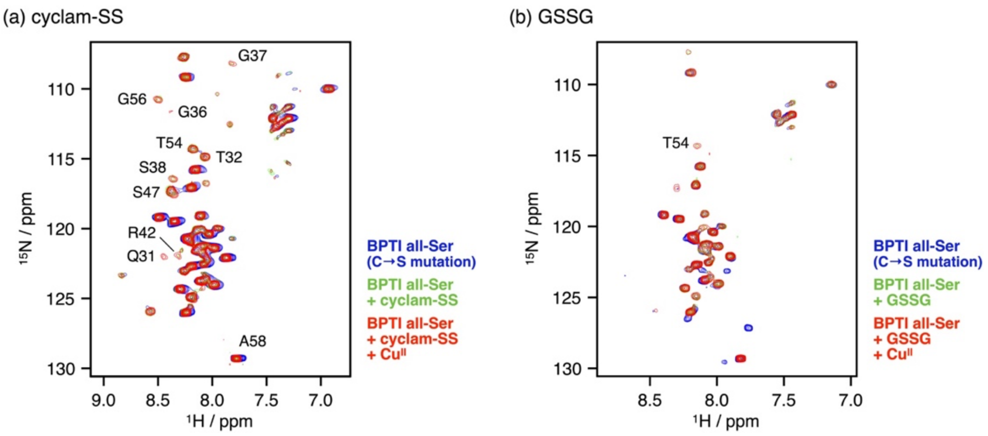

Fig. S7 ^{1}H–^{15}N correlation SOFAST-HMQC spectra of ^{15}N-labeled unfold-mimic BPTI (BPTI All-Ser) in the presence of (a) cyclam-SS or (b) GSSG. Measurement was conducted at 30 °C. [^{15}N BPTI All-Ser] = 100 μM, [cyclam-SS] = 0 or 1 mM, [GSSG] = 0 or 1 mM, [$CuCl_2$] = 0 or 1 mM, 50 mM HEPES (pH 7.0), 10 v/v% D_2O, 500 MHz.

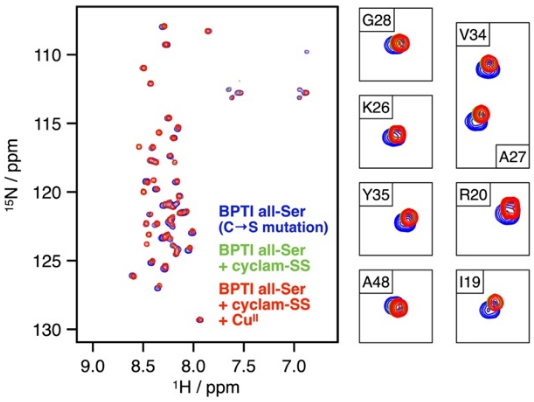

Fig. S8 ^{1}H–^{15}N correlation SOFAST-HMQC spectra of ^{15}N-labeled unfold-mimic BPTI (BPTI All-Ser) in the presence of higher concentration of cyclam-SS (5 mM). Measurement was conducted at 10 °C. Magnified views of several signals with characteristic differences are also shown. [^{15}N BPTI All-Ser] = 100 μM, [cyclam-SS] = 0 or 5 mM, [$CuCl_2$] = 0 or 5 mM, 50 mM HEPES (pH 7.0), 10 v/v% D_2O, 500 MHz.

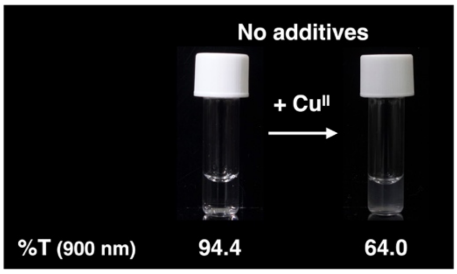

Fig. S9 Appearances of BPTI solution in the absence and in the presence of Cu^{II} ions. The turbidity (%T) of the sample solutions at 900 nm is also shown to quantitatively evaluate the BPTI aggregation. [BPTI] = 30 μM, [$CuCl_2$] = 0 or 180 μM. [urea] = 300 mM, 50 mM Tris–HCl (pH 7.5), 300 mM NaCl, 30 °C. The turbidity measurement: l = 1 cm, 30 °C.

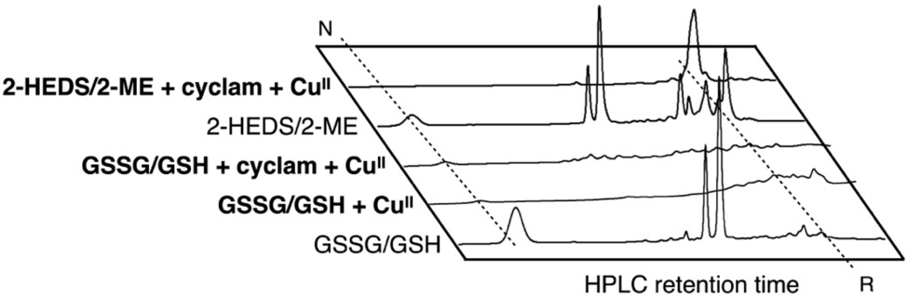

Fig. S10 HPLC analysis of BPTI folding reactions by the mixture of GSSG/GSH or 2-hydroxyethyldisulfide (2-HEDS)/2-mercaptoethanol (2-ME) in the absence and in the presence of Cu^{II} ions and the cyclam ligand. [BPTI] = 30 μM, [SS compounds] = 90 μM, [SH compounds] = 900 μM, [$CuCl_2$] = 0 or 180 μM, [cyclam] = 0, 180, or 1080 μM. [urea] = 300 mM, 50 mM Tris–HCl (pH 7.5), 300 mM NaCl, 30 °C. Eluent buffers of HPLC analyses: water (containing 0.05% TFA) and CH_3CN (containing 0.05% TFA) with a linear gradient; flow rate: 1.0 mL min^{-1}; detection wavelength: 229 nm; column temperature: 50 °C.

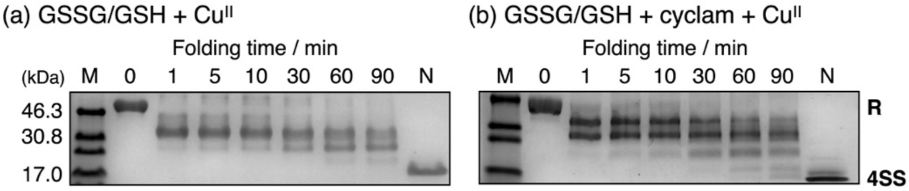

Fig. S11 SDS-PAGE analyses of RNase A oxidation by (a) GSSG/GSH and (b) GSSG/GSH and cyclam in the presence of Cu^{II} ions. [RNase A] = 8 μM, [SS compounds] = 32 μM, [SH compounds] = 320 μM, [cyclam] = 0 or 384 μM, [$CuCl_2$] = 0 or 64 μM, 50 mM Tris–HCl (pH 7.5), 300 mM NaCl, 30 °C.

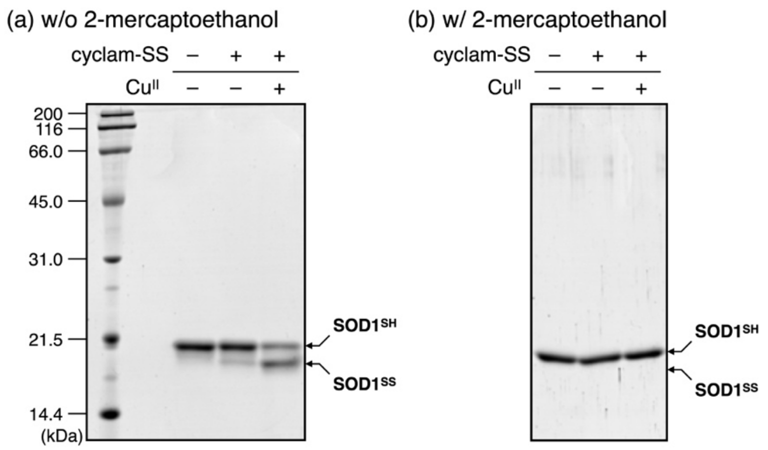

Fig. S12 SDS-PAGE analysis of E,Zn-SOD1^{SH} oxidation by cyclam-SS in the absence and presence of Cu^{II} ions. The samples were analyzed (a) without or (b) with 2-mercaptoethanol as a reductant. [SOD1] = 10 μM, [cyclam-SS] = 0 or 10 μM, [$CuCl_2$] = 0 or 10 μM, 50 mM MOPS (pH 7.0), 100 mM NaCl, 37 °C.

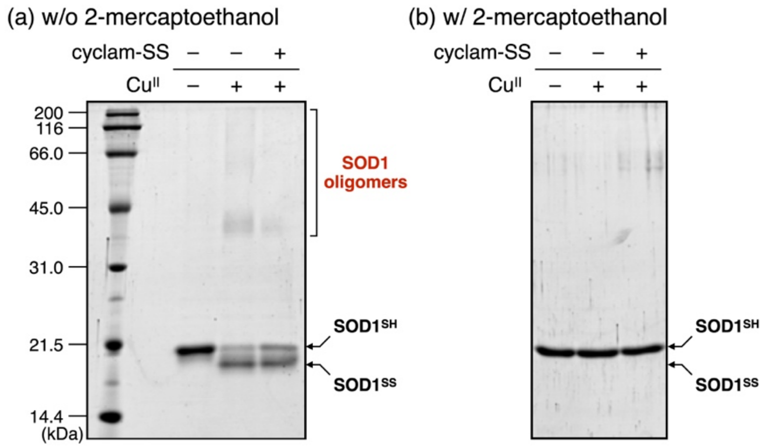

Fig. S13 SDS-PAGE analysis of Cu^{II}-dependent oligomerization of SOD1 with a G37R mutation in the absence and presence of cyclam-SS. The samples were analyzed (a) without or (b) with 2-mercaptoethanol as a reductant. [SOD1] = 10 μM, [cyclam-SS] = 0 or 10 μM, [$CuCl_2$] = 0 or 10 μM, 50 mM MOPS (pH 7.0), 100 mM NaCl, 37 °C.

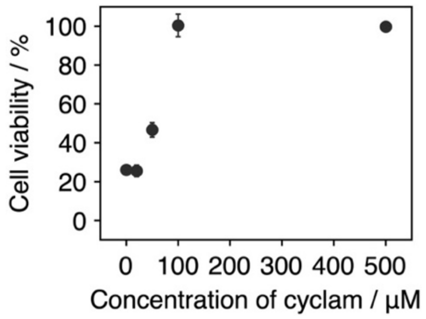

Fig. S14 Effects of cyclam on the viability of HeLa cells treated with Cu^{II} ions (100 μM). Error bars indicate the means ± SEM of three independent experiments.

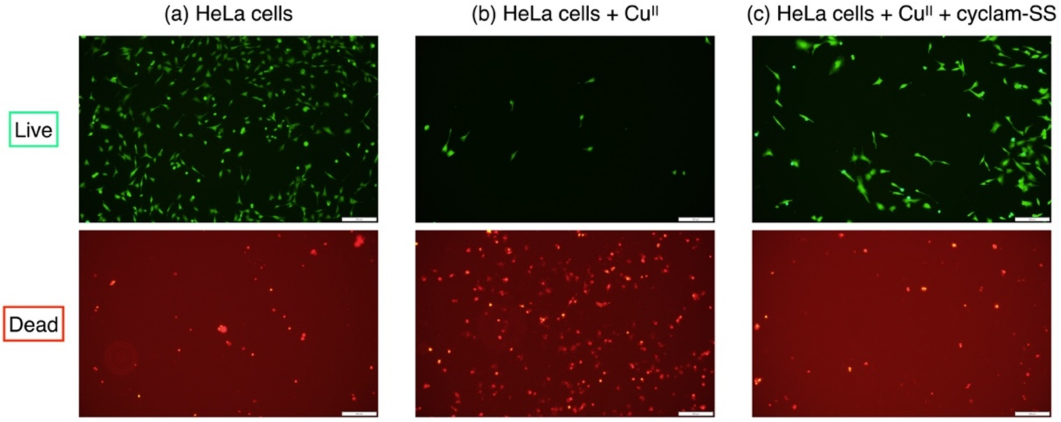

Fig. S15 Live/dead cell imaging of HeLa cells treated with Cu^{II} ions in the absence and in the presence of cyclam-SS. $[Cu^{II}]$ = 0 or 500 µM, [cyclam-SS] = 0 or 500 µM. 5000 cells were harvested and incubated at 37 °C for 24 h before the addition of Cu^{II} ions. cyclam-SS was added 4 h after the addition of Cu^{II}, and the cells were incubated for 20 h before the microscopic analysis. Scale bar = 200 µm.

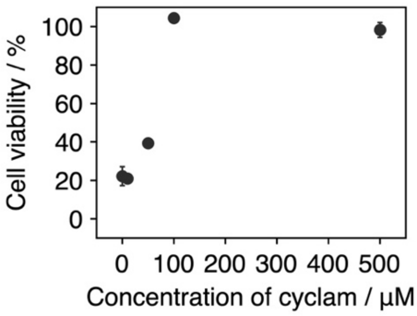

Fig. S16 Effects of the cyclam–2-hydroxyethyldisulfide mixture (2:1) on the viability of HeLa cells treated with Cu^{II} ions (100 µM). Error bars indicate the means ± SEM of three independent experiments.

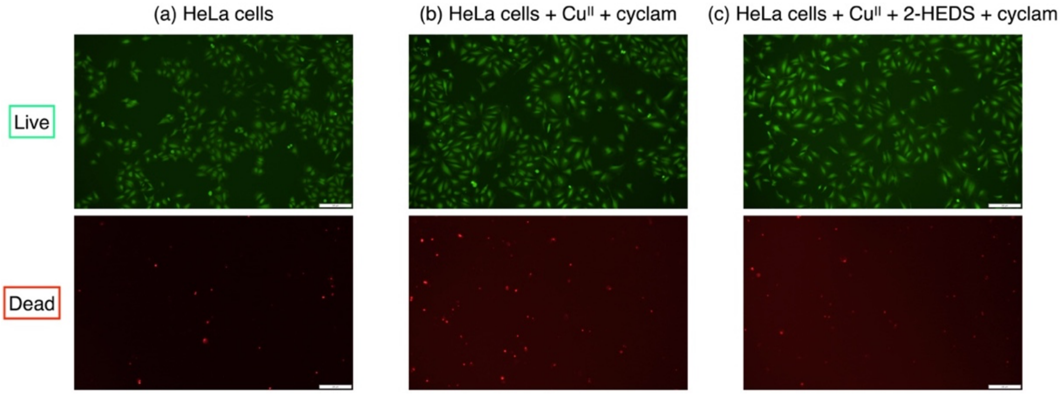

Fig. S17 Live/dead cell imaging of HeLa cells treated with Cu^{II} ions in the absence and in the presence of cyclam and 2-hydroxyethyldisulfide (2-HEDS). $[Cu^{II}]$ = 0 or 500 µM, [cyclam] = 0 or 500 µM, [2-HEDS] = 0 or 250 µM. 5000 cells were harvested and incubated at 37 °C for 24 h before the addition of Cu^{II} ions. cyclam and 2-HEDS were added 4 h after the addition of Cu^{II}, and the cells were incubated for 20 h before the microscopic analysis. Scale bar = 200 µm.

6. References

1. G. L. Ellman, *Arch. Biochem. Biophys.* **1959**, *82*, 70-77.

2. K. Arai, H. Ueno, Y. Asano, G. Chakrabarty, S. Shimodaira, G. Mugesh, M. Iwaoka, *ChemBioChem* **2018**, *19*, 207–211.

3. P. Schanda, B. Brutscher, *J. Am. Chem. Soc.* **2005**, *127*, 8014–8015.

4. G. Delaglio, S. Grzesiek, G. W. Vuister, G. Zhu, J. Pfeifer, A. Bax, *J. Biomol. NMR*, **1995**, *6*, 277-293.

5. T. Saio, K. Ishii, M. Matsusaki, H. Kumeta, S. Kanemura, M. Okumura, *bioRxiv* **2024**, preprint, DOI: 10.1101/2024.03.04.583432.

6. U. K. Laemmli, *Nature* **1970**, *227*, 680-685.

7. M. Okumura, M. Saiki, H. Yamaguchi, Y. Hidaka, *FEBS J.*, **2011**, *278*, 1137-1144.

8. T. Kuramochi, Y. Yamashita, K. Arai, S. Kanemura, T. Muraoka, M. Okumura, *Chem. Commun.* **2024**, *60*, 6134–6137.

9. Y.-H. Lee, T. Saio, M. Watabe, M. Matsusaki, S. Kanemura, Y. Lin, T. Mannen, T. Kuramochi, K. Iuchi, M. Tajiri, K. Suzuki, Y. Li, Y. Heo, Y. Kamada, K. Arai, M. Hashimoto, S. Ninagawa, Y. Hattori, H. Kumeta, A. Takeuchi, H. Abe, E. Mori, T. Muraoka, T. Okiyoneda, S. Akashi, M. Vendruscolo, K. Inaba, M. Okumura, *bioRxiv* **2024**, preprint, DOI:10.1101/2024.07.30.605722.

10. S. Erbas-Cakmak, F. P. Cakmak, S. D. Topel, T. B. Uyar, E. U. Akkaya, *Chem. Commun.* **2015**, *51*, 12258–12261.

11. C. Herrero, A. Quaranta, S. E. Ghachtouli, B. Vauzeilles, W. Leiblb, A. Aukauloo, *Phys. Chem. Chem. Phys.* **2014**, *16*, 12067–12072.

12. K. K. Millis, K. H. Weaver, D. L. Rabenstein, *J. Org. Chem.* **1993**, *58*, 4144–4146.
